# Thymosin β4 released from functionalized self-assembling peptide activates epicardium and enhances repair of infarcted myocardium

**DOI:** 10.7150/thno.52309

**Published:** 2021-02-20

**Authors:** Yong-li Wang, Shu-na Yu, Hao-ran Shen, Hai-jie Wang, Xue-ping Wu, Qiang-li Wang, Bin Zhou, Yu-zhen Tan

**Affiliations:** 1 Department of Anatomy, Histology and Embryology, Shanghai Medical School of Fudan University, Shanghai 200032, China; 2 Laboratory of Oral Microbiota and Systemic Diseases, Shanghai Ninth People's Hospital, College of Stomatology, Shanghai Jiao Tong University School of Medicine, Shanghai 200125, China.; 3 School of Basic Medical Sciences, Shanghai University of Traditional Chinese Medicine, Shanghai 201203, China.; 4 Shanghai Institute of Biochemistry and Cell Biology, Chinese Academy of Sciences, Shanghai 200032, China

**Keywords:** self-assembling peptide, thymosin beta 4, epicardium, epicardium-derived cells, myocardial infarction

## Abstract

The epicardium plays an important role in cardiomyogenesis during development, while it becomes quiescent in adult heart during homeostasis. This study investigates the efficiency of thymosin β4 (Tβ4) release with RPRHQGVM conjugated to the C-terminus of RADA16-I (RADA-RPR), the functionalized self-assembling peptide (SAP), to activate the epicardium and repairing the infarcted myocardium.

**Methods:** The functionalized SAP was constituted with self-assembling motif, Tβ4-binding site, and cell adhesive ligand. Myocardial infarction (MI) models of the transgenic mice were established by ligation of the left anterior descending coronary artery. At one week after intramyocardial injection of Tβ4-conjugated SAP, the activation of the epicardium was assessed. At four weeks after implantation, the migration and differentiation of epicardium-derived cells (EPDCs) as well as angiogenesis, lymphangiogenesis and myocardial regeneration were examined.

**Results:** We found that the designer RADA-RPR bound Tβ4 and adhered to EPDCs and that Tβ4 released from the functionalized SAP could effectively activate the epicardium and induce EPDCs to differentiate towards cardiovascular cells as well as lymphatic endothelial cells. Moreover, SAP-released Tβ4 (SAP-Tβ4) promoted proliferation of cardiomyocytes. Furthermore, angiogenesis, lymphangiogenesis and myocardial regeneration were enhanced in the MI models at 4 weeks after delivery of SAP-Tβ4 along with attenuation of adverse myocardial remodeling and significantly improved cardiac function.

**Conclusions:** These results demonstrate that sustained release of Tβ4 from the functionalized SAP can activate the epicardium and effectively enhance the repair of infarcted myocardium. We believe the delivery of SAP-Tβ4 may be a promising strategy for MI therapy.

## Introduction

After the occlusion of a coronary artery or its branches, the resulting ischemia can be severe leading to the death of both cardiomyocytes and non-myocytes locally, followed by inflammation, degradation of extracellular matrix, scar formation and eventually, remodelling of the ventricular wall. In most extreme cases, the patients may die of heart failure or arrhythmia. The heart with myocardial infarction (MI) may lose up to one billion cardiomyocytes, accounting for approximately 25% of its mass 1. Although MI mortality has declined following modern conventional therapies including pharmacotherapy, percutaneous coronary intervention and coronary artery bypass grafting, MI remains a leading cause of death worldwide 2. In the recent years, stem cell-based regenerative therapies have been tested preclinically and clinically, though many cell types were ultimately found to be ineffective 3. Notably, the survival and differentiation of the engrafted cells are poor within the hostile ischemic and inflammatory microenvironment 4. While the mammalian heart appears capable of endogenous regeneration at the early neonatal stage, this capacity is lost shortly after 5-6. The myocardium of neonatal mice is regenerated in part after partial surgical resection of ventricular apex 7. Intriguingly, the newborn human heart may also have intrinsic capacity to repair myocardial damage and completely recover cardiac function 8. In human heart, renewal of cardiomyocytes is up to 1% per year at the age of 25 to 0.45% at the age of 75 9. Therefore, it is important for effective repair of the infarcted myocardium to reactivate or enhance the endogenous cardiac regenerative potential.

Recent studies have suggested that the epicardium plays an important role in heart development as well as regeneration of the injured myocardium 10. The epicardium, the visceral layer of the serosal pericardium, covers the heart and the juxta-cardiac parts of its greater vessels. The subepicardium, on the other hand, is composed mostly of loose connective tissues. During development, the epicardium-derived cells (EPDCs) contribute to vascularization and formation of the interstitium in the heart 11. Moreover, the EPDCs may, in rare cases, contribute to the cardiomyocyte lineage 12, though the extent of this contribution is unclear 10. However, the epicardium becomes quiescent in the normal adult heart 13. In the injured zebrafish heart, EPDCs migrated into the subepicardium and the myocardial interstitium to generate endothelial and smooth muscle cells of the vasculature 14. After MI, the EPDCs of the adult mice may contribute to terminally differentiated cardiomyocytes in a limited fashion 15. It has been shown that EPDCs in the infarcted mouse heart differentiated towards fibroblasts, myofibroblasts, and endothelial cells, and regulated the regeneration of cardiomyocytes, however the number of newly generated cardiomyocytes were insufficient for replacing the number of lost cardiomyocytes 16. Although epicardium-mediated cardiac regeneration occurs more efficienly in some vertebrates, the human myocardium is incapable of adequate regeneration post-MI. Thus, it is necessary to explore optimal strategies for activating the epicardium and promoting EPDC differentiation.

In recent years, thymosin β4 (Tβ4) has received significant attention for its ability to enhance cardiac regeneration. Tβ4, a G-actin-sequestering peptide, is expressed in the embryonic heart 17. Heart-specific Tβ4 knockdown results in epicardial and myocardial defects as well as abnormal coronary artery development 18. In the injured adult heart, Tβ4 expression is up-regulated during the early stages of regeneration 19. Tβ4 can reactivate epicardial embryonic genes such as Wilms tumor 1 (WT1) and T-box factor 18 (Tbx18) 20. The activated epicardial cells can undergo epithelial to mesenchymal transition (EMT) 21 that results in their proliferation, migration towards the subepicardium and myocardium of the injury site, and differentiate into cardiovascular cells 22. Tβ4-primed adult heart exhibits augmentation of embryonic reprogramming in the epicardium, promoting neovascularization and cardiomyogenesis post-MI 15, 23. Furthermore, Tβ4 promotes migration and differentiation of EPDCs 18, 24. Given these salubrious properties of Tβ4, recent studies have addressed the boosting of pro-regenerative epicardial properties of the injured heart by applying exogenous Tβ4. Tβ4 has been administered by intraperitoneal injection 25, 26 or local release with collagen-chitosan hydrogel 27 for repairing the injured myocardium following experimental MI or ischemia-reperfusion injury. However, these approaches to deliver Tβ4 apparently do not effectively activate the epicardium for cardiac injury repair.

The current study demonstrates the synthesis of a functionalized self-assembling peptide (SAP) through molecular simulation and examined the effectiveness of sustained Tβ4 release from the functionalized SAP to enhance epicardial activation. Activation of the epicardium was identified via their re-expression of a master embryonic epicardial gene WT1. WT1 is expressed in the proepicardium as well as adult injured epicardium and EPDCs 28, 29. Migration and differentiation of EPDCs were monitored based on their expression of WT1 using a genetic EPDC-labelling strategy. Here, we demonstrate that the functionalized SAP can provide a sustained release of Tβ4 *in vivo*. This sustained-released Tβ4 promotes the survival, proliferation, and migration of EPDCs, and their differentiation towards vascular and lymphatic endothelial cells, smooth muscle cells and cardiomyocytes. After injection of SAP-Tβ4 into the MI heart, we noted the dramatic enhancement of neovascularization, lymphangiogenesis, and myocardial regeneration in the infarcted myocardium. Thus, our study demonstrates that the delivery SAP-Tβ4 can activate the epicardium and promote the effective repair of the infarcted myocardium.

## Methods

### Establishment of MI models

This investigation was approved by the Institutional Animal Care Committee of Fudan University. WT1^CreERT2/+^; Rosa26^mTmG/+^ transgenic mice were kindly provided by Shanghai Institute of Biochemistry and Cell Biology, Chinese Academy of Sciences. In the mice, a Cre-modified oestrogen ligand-binding domain (CreERT2) is knocked into the WT1 locus. Activity of CreERT2 fusion protein recombinase is triggered by tamoxifen binding. Then, the *WT1*-expressing cells in epicardium are labeled with membrane-localized GFP and simultaneously inactivated expression of membrane-localized dTomato fluorescent protein. These epicardial GFP^+^ cells, consisting of *WT1*-expressing cells and their descendants, are defined as EPDCs. MI models of the WT1^CreERT2/+^; Rosa26^mTmG/+^ transgenic mice (male, 12 weeks) were established according to the procedure previously described 30. Before establishment of MI models, tamoxifen (0.2 mg/g body weight) was intraperitoneally injected twice each week for 3 weeks to trigger lineage tracing. At 1 week after tamoxifen administration, 108 mice (20 - 25 g) were anaesthetized with 2% isoflurane. After endotracheal intubation and ventilation, thoracotomy was performed at the left third intercostal space. The pericardium was opened, and then the left anterior descending coronary artery (LAD) was ligated with 7-0 silk suture. Successful establishment of MI models was determined by observing a pale discoloration of the myocardium and a ST-elevation in the electrocardiogram. Sham-operated mice underwent the same surgery without the coronary artery ligation.

### Isolation and identification of EPDCs

EPDCs were isolated from the hearts at 1 week after MI as previously described 31. Briefly, the hearts were removed and then digested with mixed solution of 0.05% trypsin (Invitrogen, Carlsbad, CA, USA) and 0.08% collagenase IV (Sigma-Aldrich, Saint Louise, MO, USA) in a 37 °C rocking water bath. The supernatant was removed every 6 min and neutralized with fetal bovine serum (FBS; Invitrogen). After digestion for six times, the dissociated cells were filtrated through 70-μm cell strainer (Millipore Corporation, Billerica, MA, USA) followed by centrifugation at 200 g for 5 min. The cells were incubated with Dulbecco's modified Eagle's medium (DMEM; Invitrogen) supplemented with 20% FBS in gelatin-coated dishes for 1 h. After the non-adherent cells were removed by gentle washing, the adherent cells were continued to be incubated. When being grown into confluence, the cells were trypsinized and passaged. For sorting of EPDCs, the second or third passage of the cells were harvested and suspended with DMEM supplemented with 2% FBS. GFP^+^ EPDCs were sorted using a fluorescence-activated cell sorter (FACS; Beckman Coulter, Fullerton, CA, USA).

For identifying EPDCs, expression of WT1 and Tbx18 in the sorted cells was examined with immunostaining, and expression of *WT1*, *Tbx18*, *Tcf21* (*transcription factor 21*), *cTnT* (*cardiac troponin T*), *CD31* and *CNN1* (*calponin 1*) in the cells was assessed with quantitative reverse transcription-polymerase chain reaction (qRT-PCR). For immunostaining, the cells were incubated with rabbit anti-WT1 (1:100, Santa Cruz, Dallas, TX, USA), rabbit anti-Tbx18 (1:100; Abcam, Cambrige, MA, USA) overnight at 4^ o^C. Then, the cells were incubated with Alexa Fluor 594-conjuncted goat anti-rabbit IgG (1:400; Jackson, West Grove, PA, USA) for 30 min at 37^ o^C. For qRT-PCR, total RNA was extracted from the sorted cells with the trizol reagent (Invitrogen). RNA was reverse-transcribed to first-strand cDNA by using a PrimeScript reverse transcriptase reagent kit (Takara, Otsu, Japan). qRT-PCR was performed on StepOnePlus Real-Time PCR System (Applied Biosystems, Carlsbad, CA, USA) with the following cycle conditions: 95^ o^C for 30 s, followed by 40 cycles of 95^ o^C for 15 s, 60^ o^C for 30 s, and 72^ o^C for 30 s. SYBR premix Ex Taq (Takara) was used in each reaction. The mRNA levels in relation to 28S rRNA were determined by 2^-ΔΔCt^ method and normalized the gene expression levels to β-actin levels. The myocardium was used as the control. The sequences of the primers were shown in Table S1.

### SAP synthesis

RADA16-I was modified with a bioactive short-peptide motif, which was screened from several short peptides (DDETTDDE, DEDETT, HQRHQGHQ, PIYEGYA, and RPRHQGVM). To understand the binding mode of Tβ4 and these short peptides, a docking study was performed using Surflex-Dock in Sybyl version 8.02 operating under Red Hat Linux 4.0 32. According to the results of Surflex-Dock, RPRHQGVM was selected, and then RPRHQGVM combined with RGDS was conjugated to the C-terminus of RADA16-I to obtain a novel functionalized SAP (RADA-RPR; Table S2). RADA-RPR was synthesized by Top-Peptide Biotechnology Company (Shanghai, China). Molecular weight and purity of the peptide were examined with a mass spectrometer and an ultra-performance liquid chromatography (Agilent Technologies, Santa Clara, CA, USA) respectively.

### Atomic force and scanning electron microscopies

For assessing self-assembly, RADA-RPR was dissolved in Milli-Q water (18.2 MΩ) at a concentration of 1% (10 mg/mL). After sonicating for 30 min, the solution was diluted to a working concentration (0.01%). 5 µL of the sample was dropped onto freshly cleaved mica at 30 min and 2 h respectively after sonication. The mica was air-dried, and examined with an atomic force microscope (AFM; Bruker Corporation, Billerica, MA, USA). The images were obtained by scanning the mica surface in tapping mode. In scanning electron microscopy, 1% RADA-RPR solution was diluted with PBS (1:4), and allowed to gelate at 37 °C for 1 h. After fixing, dehydrating and coating with platinum, the nanofiberous scaffold of the gel was examined using a scanning electron microscope (SEM; Philips, DA Best, The Netherlands).

### Immunogenicity detection of the functionalized SAP

To evaluate the immunogenicity of the functionalized SAP, 100 µL peptide solution was injected into the subcutaneous tissue at the back of C57BL/6 mice at 5 spots. The same amount of PBS was injected as the control. After 1 week, all the mice were anesthetized with ketamine, and the skin at the injected region was removed. After fixation with 4% paraformaldehyde solution at 4 °C overnight, the tissue was dehydrated with 15% and 30% sucrose phosphate buffer gradually, and then cryosections were prepared. After immunostaining, expression of CD68 and CCR7 was used to detect aggregation of immune cells such as macrophages and T lymphocytes.

### Ultra-performance liquid chromatography-tandem mass spectrometry (UPLC-MS/MS)

To detect Tβ4 released from SAP hydrogel, UPLC-MS/MS was performed. 1 µg Tβ4 (ProSpec, East Brunswick, NJ, USA) was fully blended with 20 µL peptide solution for 5 min. Then, 80 µL PBS were added to trigger gelation of the peptide. After equilibrating the pH, 1 mL PBS was added onto the gel. The hydrogel was incubated at 37^ o^C. 20 µL of the supernatant was collected at 1 d, 3 d, 7 d, 14 d, 21 d and 28 d respectively. Concentration of Tβ4 in the supernatant was detected by ultra-performance liquid chromatograph and mass spectrometer (Agilent Technologies). Agilent Mass Hunter B4.0 software was used for data acquisition and analysis. The cumulative profile of Tβ4 release was drawn. Tβ4 reference standards (100 ng/mL, 200 ng/mL, 400 ng/mL, 600 ng/mL, 800 ng/mL and 1000 ng/mL) were injected into the column respectively as the controls.

### Treatment with hypoxia and serum deprivation

To evaluate effect of Tβ4 released from the functionalized SAP on EPDC survival, EPDCs were divided into control, Tβ4, SAP and SAP-Tβ4 groups. The cells (1 × 10^5^/mL) were seeded in the 6-well plates. In Tβ4 group, Tβ4 (1 µg/mL) was added into the medium. In SAP and SAP-Tβ4 groups, 20 µL peptide or 20 µL peptide containing 1 µg Tβ4 solution were mixed with 80 µL PBS. Then, the cells were seeded on the gels in 6-well plates. After incubation for 24 h, the medium was changed with fresh DMEM. When grown to 80% confluence, the cells were incubated in the condition of hypoxia (1% O_2_) and serum-free for 12 h. Concentration of lactate dehydrogenase (LDH) in the supernatant was detected with a LDH ELISA kit (Guchen Biotechnology, Shanghai, China). Absorbance at 450 nm was measured with Infinite 200 PRO Microplate Reader (Tecan, Mannedort, Switzerland). Apoptosis of the cells was examined with EB/AO (ethidium bromide/acridine orange) staining. Percentages of the apoptotic cells in five random fields were calculated. The assay was repeated five times.

### Proliferation assay

The effect of Tβ4 released from SAP on EPDC proliferation was evaluated by Ki67 immunostaining and cell counting kit-8 (CCK-8) assay. The cells were divided into four groups as above. In Ki67 immunostaining, the cells (1 × 10^5^/mL) were incubated in DMEM supplemented with 15% FBS for 3 days, and then incubated with rabbit anti-Ki67 antibody (1:200, Abcam) and Alexa Fluor 594-labelled goat anti-rabbit IgG. Ki67-positive cells were viewed with a fluorescent microscope. Percentage of the Ki67-positive cells in total cells was calculated using an Image-Pro Plus 6.0 software (Media Cybernetics, Bethesda, MD, USA). In CCK-8 assay, the cells (4 × 10^3^/well) were incubated for 3 days or 7 days in the 96-well plate. Then, 10 µL of CCK-8 (Dojindo, Kumamoto, Japan) was added to each well, and the cells continued to be incubated for 2 h. The absorbance at 450 nm was measured using Infinite 200 PRO Microplate Reader. Five repeated experiments were performed for each group.

### Migration assay

The experimental grouping is the same as above. The cells (3 × 10^5^/mL) were seeded in petri dishes. When the cells were grown to confluence, a straight scratch was made with the tip of a p200 pipette. After washing with PBS, the cells were incubated with fresh DMEM for 16 h. The areas of cells migrated into the scratched region were measured using Image-Pro Plus 6.0 software. To assess expression of the genes related to cell motility, total RNA of the cells was extracted at 16 h after the scratch. Expression of *myosin light chain kinase* (*Mylk*), *Rho-associated protein kinase 1* (*Rock1*) and *vinculin* (*Vcl*) were analyzed with qRT-PCR.

### Induction of EPDC differentiation

The cells (1 × 10^5^/mL or 3 × 10^5^/mL) were treated as above for assessing differentiation towards vascular cells and cardiomyocytes. After induction for 24 h, the medium was changed, and the cells continued to be incubated. Expression of endothelial cell marker genes *CD31* and *vWF* (*von Willebrand factor*), smooth muscle cell marker genes *α-SMA* (*α-smooth muscle actin*) and *CNN1*, early myocardial transcription factor *Nkx2.5* and *GATA4* (at 1 week) and cardiac-specific genes *cTnT* and* Cx43* (*connexin-43*; at 2 weeks) were analyzed with qRT-PCR. Moreover, expression of CD31, α-SMA (at 2 weeks) and cTnT (at 4 weeks) was examined with immunostaining. The cells were incubated with mouse anti-CD31 (1:100) and mouse anti-α-SMA (1:100, Abcam) or mouse anti-cTnT (1:200, Santa Cruz) overnight at 4 °C. After washing with PBS, the cells were incubated with Alexa Fluor 594-conjuncted goat anti-mouse IgG (1:400) for 30 min at 37 °C. The nuclei were counterstained with DAPI (4', 6-diamidino-2-phenylindole, 1:1000; Sigma). Expression of CD31, α-SMA and cTnT in the cells differentiated from GFP^+^ EPDCs was examined using a fluorescence microscope.

### Western blotting

cTnT expression of the cells at 4 weeks after induction was assessed with Western blotting. The cells were lysed with RIPA buffer (Beyotime, Beijing, China). After quantifying with BCA protein assay kit (Beyotime), equal amount of proteins were separated on 15% SDS-PAGE and then transferred onto PVDF membranes. Non-specific binding sites were blocked using 5% skim milk. The membranes were incubated with mouse anti-cTnT antibody (1: 1000) or mouse anti-β-actin monoclonal antibody (1: 4000; Peprotech, Rocky Hill, NJ, USA) at 4 °C overnight, and incubated with horseradish peroxidase-conjugated goat anti-mouse IgG (1:4000; Cell Signaling, Danvers, MA, USA) for 1 h at room temperature. Then, the membranes were visualized with the enhanced chemiluminescence. β-actin was used as an internal control for protein loading. Relative expression of cTnT was shown as a ratio of cTnT/β-actin.

### Enzyme-linked immunosorbent assay (ELISA)

The effect of Tβ4 released from SAP on paracrine of EPDCs was evaluated by ELISA. At 1 week after incubation in DMEM containing 15% FBS, basic fibroblast growth factor (bFGF), hepatocyte growth factor (HGF), insulin-like growth factor 1 (IGF-1), platelet-derived growth factor BB (PDGF-BB), stem cell factor (SCF), stromal cell-derived factor 1 (SDF-1), transforming growth factor β1 (TGF-β1) and vascular endothelial growth factor (VEGF) in the supernatant were detected with ELISA Kit (Guchen Biotechnology), absorbance values were measured with a microplate reader (Tecan).

### Implantation of the SAP carrying Tβ4

At 1 week after LAD ligation, more than 60% mice survived, and regarded as successful MI models. The survived mice were randomly divided into sham operation (n = 8), control (n = 9), Tβ4 (n = 9), SAP (n = 9) and SAP-Tβ4 (n = 10) groups. The second thoracotomy was performed. Before injection, the peptide solution was sonicated for 30 min. In SAP-Tβ4 group, 20 µL peptide solution and 10 µg Tβ4 were fully blended for 5 min, and then mixed with PBS to 60 µL. The mixture was intramyocardially injected at four sites (15 µL per site) of peri-infarct region. In Tβ4 and SAP groups, the same dosages of Tβ4 or SAP were injected. In sham operation and control groups, the same volume of PBS was injected.

### Echocardiography

Cardiac function of all mice was recorded in normal mice, before MI, 1 week after MI and 4 weeks after intramyocardial injection using an echocardiographic machine (VisualSonics, Toronto, Canada) with a 30 MHz linear transducer. Left ventricular end-systolic internal diameter (LVESD) and LV end-diastolic internal diameter (LVEDD) were determined in M-mode images with a well-defined continuous interface between the septum and posterior wall. For examining systolic function, LV end-diastolic volume (LVEDV), LV end-systolic volume (LVESV), ejection fraction (EF = LVEDV - LVESV/LVEDV × 100%), and fractional shortening (FS = LVEDD - LVESD/LVEDD × 100%) were measured. Two echocardiographers blinded to the experimental treatment acquired the images. Three measurements at least were taken and averaged for each parameter.

### Histologic examination of the hearts

At 4 weeks after implantation, all hearts were harvested and fixed by perfusion with 4% paraformaldehyde solution. Then, the hearts were cut into upper and lower parts with cross-section and continued to be fixed with paraformaldehyde solution at 4°C overnight. After washing, the tissue was dehydrated with 15% and 30% sucrose phosphate buffer gradually, and then embedded in Tissue-Tek OCT (optimal cutting temperature compound; Sakura Finetek, Torrance, CA, USA). To assess fibrosis of the myocardium, 5 µm-thick cryosections were prepared and stained with Masson's trichrome. Collagen-rich scar tissue was stained blue, while myocardial tissue was stained red. The thickness of LV wall was measured at the minimum thickness region of the infarcted LV wall, and the scar size was calculated as percentage of circumference of the infarct region in whole LV wall circumference.

### Activation of the epicardial cells and tracing of GFP^+^ EPDCs

In order to evaluate the effect of Tβ4 released from SAP on activating the epicardial cells, the mice at 1 week after MI were divided in control, Tβ4, SAP and SAP-Tβ4 groups (n = 5). At 1 week after implantation, the hearts were harvested, and cryosections were prepared as above. Activation of the epicardial cells and expression of Tβ4 in the epicardium and myocardium at the infarct region were determined by GFP or Tβ4 and CD31 double immunostaining.

To trace differentiation of GFP^+^ EPDCs into vascular and lymphatic endothelial cells, smooth muscle cells and cardiomyocytes, double immunostaining of GFP and CD31, LYVE-1 (lymphatic vessel endothelial hyaluronan receptor 1), a-SMA or cTnT was performed. The junction between the cardiomyocyte differentiated from EPDCs with resident cardiomyocyte was identified by triple-labelled immunostaining of GFP, cTnT and Cx43. Densities of the microvessels and lymphatic vessels were assessed by counting CD31^+^ vessels in the infarct region and measuring area of the transverse sections of LYVE-1^+^ vessels in the peri-infarct region with ImageJ 1.46r software (Wayne Rasband, NIH, USA) respectively. At least 5 independent sections and 2 fields (20 x) on one section were selected randomly.

### Immunostaining of cardiac tissues

The cryosections were incubated with Tβ4 and GFP (1:50; Santa Cruz), CD31, α-SMA, cTnT and Cx43 (1:100; Cell Signalling), and LYVE-1 (1:100; Novus) antibodies at 4 °C overnight. Then, the sections were incubated with Alexa Flour 488 conjugated goat anti-rabbit IgG (1:300; Jackson) and Alexa Fluor 594 conjugated goat anti-mouse IgG (1:400; Jackson) or DyLight 488 conjugated goat anti-chicken IgG (1:400; Novus), DyLight 594 conjugated goat anti-rabbit IgG (1:400; Abcam) and Alexa Fluor 647 conjugated goat anti-mouse IgG (1:400; Jackson) at room temperature for 1 h. The antibodies used for immunostaining were described in Table S3.

For assessing cardiomyocyte proliferation, double immunostaining of cTnT and Ki67 (1:200) or Aurora B (1:100, Sigma-Aldrich) was performed. cTnT^+^Ki67^+^ and cTnT^+^Aurora B^+^ cells were considered as proliferating cardiomyocytes. The number of cTnT^+^Ki67^+^ and cTnT^+^Aurora B^+^ cells in each field (20×) was calculated as above.

Expression of *bFGF*, *HGF*, *HIF-1α* (*hypoxia-inducible factor 1-α*), *IGF-1*, *PDGF-BB*, *SCF*, *SDF-1* and *VEGF* in the infarcted myocardium was assessed by qRT-PCR. Total RNA was extracted from the tissues of the ventricular wall at 1 week after implantation (three mice for each group).

### Statistical analysis

Data were expressed as mean ± SD and analyzed using GraphPad Prism (version 6.0, La Jolla, CA, USA). To analyze the data statistically, Student's *t*-test and one-way analysis of variance were performed with Scheffe's *post hoc* multiple comparison analysis. A value of *p* < 0.05 was considered as statistically significant.

## Results

### Characterization of EPDCs

**Figure 1:**
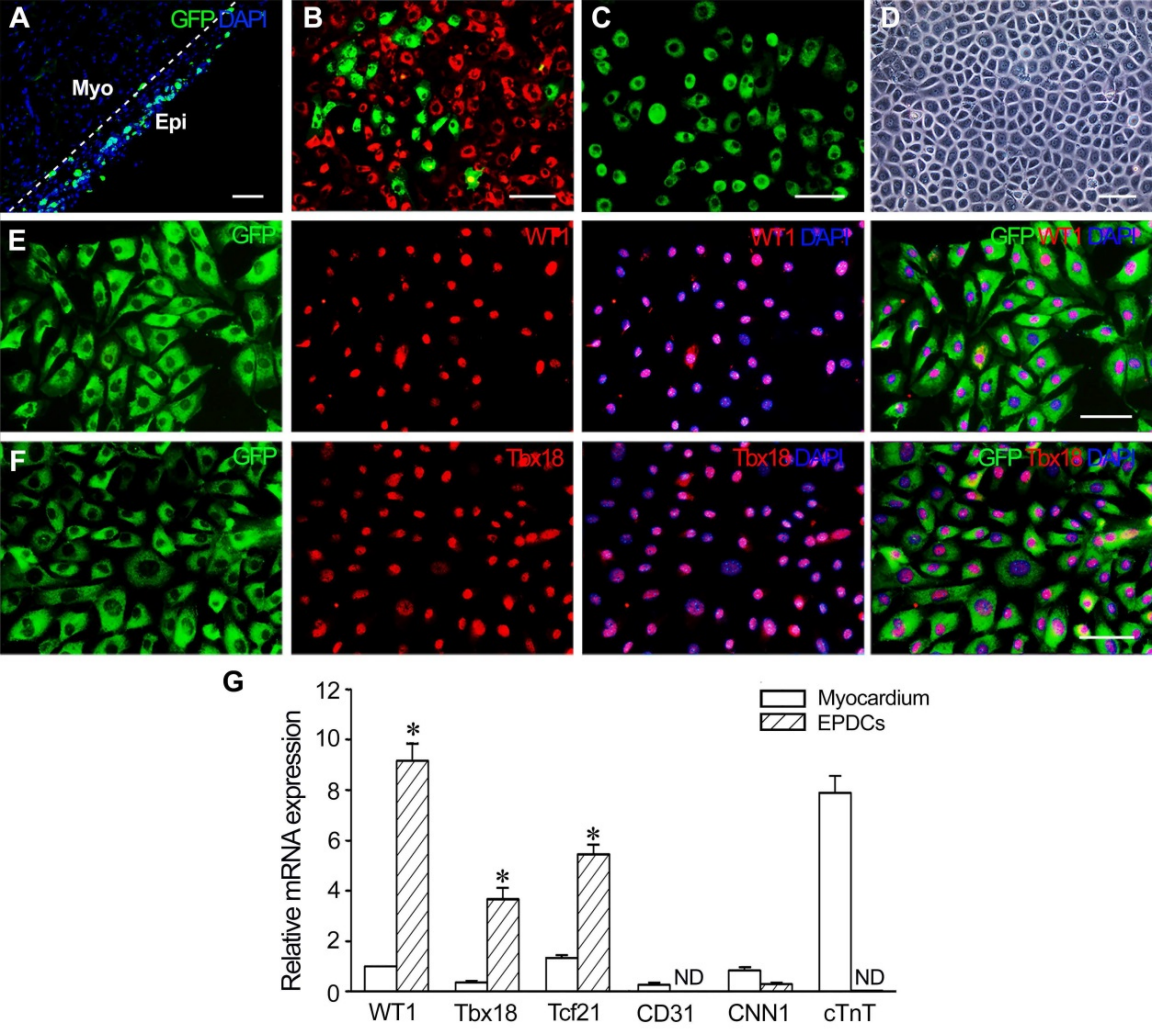
** Characteristics of EPDCs isolated from the transgenic mice at 1 week post-MI. (A)** The epicardium at 1 week post-MI. The expression of GFP represents activated epicardial cells. The dotted line indicates the junction between the epicardium (Epi) and myocardium (Myo). **(B)** The cells isolated from the epicardium. Note the presence of activated EPDCs (GFP^+^ cells). The cells with red fluorescence (dTomato^+^ cells) are epicardial cells that are not activated. **(C)** Sorted EPDCs using flow cytometry. **(D)** Phase contrast image of a monolayer of EPDCs. **(E)** Expression of WT1 in EPDCs. **(F)** Expression of Tbx18 in EPDCs. Immunostaining. Scale bar = 50 µm (A-C), 20 µm (D-F). **(G)** Expression of *Wt1*, *Tbx18*, *Tcf21, cTnT*, *CD31* and *CNN1* in the sorted cells. qRT-PCR analysis. ND, not detected. *p < 0.01 versus myocardium. n = 4.

At 1 week after MI, the epicardium of the WT1^CreERT2/+^/ROSA26^mTmG/+^ transgenic mice became thickened and expressed GFP specifically (Figure 1A), which represents the activation of endogenous WT1 expression. There are 29.2% GFP^+^ EPDCs among cells isolated from the epicardium (Figure 1B). After incubation for 48 h, the sorted GFP^+^ cells (Figure 1C) were grown into monolayer, which displayed an epithelial-like morphological feature (Figure 1D). The results of immunostaining showed that the cells expressed WT1 and Tbx18 (Figure 1E and F). Furthermore, these cells expressed *WT1*, *Tbx18* and *Tcf21* specifically. However, no expression of *CD31*, *CNN1* and* cTnT* was observed, which indicated that the sorted cells were not contaminated with endothelial cells, smooth muscle cells or cardiomyocytes (Figure 1G).

### Design of the functionalized SAP

**Figure 2:**
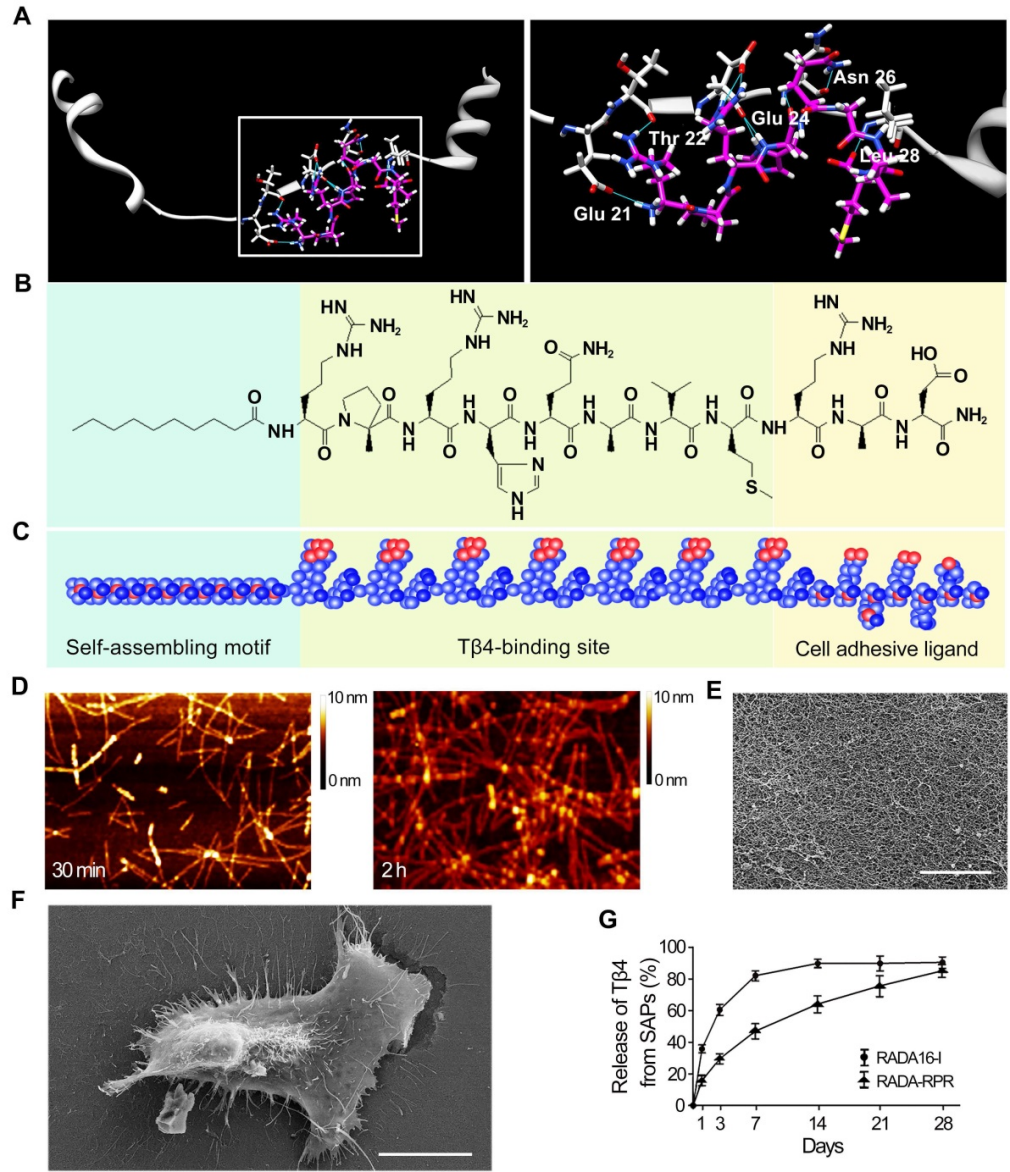
** The features of the designer functionalized SAP and the sustained release of Tβ4 from the SAP. (A)** The mode of the binding site docked into Tβ4. White, Tβ4; Pink, Tβ4-binding site; Blue, hydrogen bonds. The right panel is magnification of the box in the left panel. **(B, C)** The chemical structure (B) and molecular model (C) of the SAP. It contains self-assembling motif, Tβ4-binding site and cell adhesive ligand. **(D)** The nanofibers formed by spontaneous assembling of RADA-RPR peptide at 30 min and 2 h after sonication. AFM images. **(E)** The scaffolds organized by SAP nanofibers. SEM image. Scale bar = 2 µm. **(F)** A cell spread on the SAP scaffolds. SEM image. Scale bar = 10 µm. **(G)** The cumulative release profile for Tβ4 from RADA-RPR. n = 3.

Surflex-Dock was applied to study molecular docking of Tβ4 and Tβ4-binding site. After running Surflex-Dock, 9 hydrogen bonds were predicted, and the detailed binding patterns in the cavity were speculated. Figure 2A showed hydrogen bonding interactions between Tβ4 (consisting of acidic residue Glu21, Glu24 and neutral residue Thr22, Asn26, Leu28) and Tβ4-binding site (RPRHQGVM). Moreover, the types of the hydrogen bonds included C = O…H-N, H-N…H-N, C-O…H-N, H-O…H-N and C = O…H-O. As shown in Figure 2A, hydrophobic interactions are established between alkyl groups, carbocyclic ring and hydrophobic residues. Surflex-Dock score was 6.71. The score indicated that binding affinity of Tβ4 with Tβ4-binding site was strong. A schematic illustration of the designer functionalized SAP is shown in Figure 2B and C. It was constituted with self-assembling motif, Tβ4-binding site and cell adhesive ligand.

### Characteristics of the SAP

The purity of RADA-RPR was > 96%. Its relative molecular weight was consistent with the theoretical value (data not shown). After sonication for 30 min, the peptide was self-assembled to form nanofibers with a diameter of 5 to 10 nm and a length of 100 to 300 nm (Figure 2D). At 2 h after sonication, the peptides formed a uniform and interwoven nanofibrous scaffold (Figure 2D and E). The cells spread out well on the nanofibrous scaffold (Figure 2F). In addition, immunostaining of the subcutaneous tissue showed that the difference in the number of CD68^+^ or CCR7^+^ immune cells between SAP group and control group was not significant (Figure S1). After implantation, the SAP remained for 4 weeks in the myocardium (data not shown).

### The sustained release of Tβ4 from the SAP hydrogel

Concentration of Tβ4 in the supernatant from RADA-RPR hydrogel showed an initial rapid release in the first 3 days followed by a steady release that persisted over 28 days. Compared with RADA16-I hydrogel, the RADA-RPR hydrogel showed slower and more sustained release of Tβ4 (Figure 2G).

### Effect of SAP-released Tβ4 on survival of EPDCs

In hypoxic condition with serum deprivation, there were fewer apoptotic cells in the Tβ4 and SAP groups than those in control group. The number of the apoptotic cells was significantly lower in the SAP-Tβ4 group compared with Tβ4 and SAP groups (Figure S2A-C). The concentrations of LDH in the supernatant in Tβ4 and SAP groups were decreased significantly compared with control while the concentration of LDH was significantly lower in the SAP-Tβ4 group than in the Tβ4 group (Figure S2D).

### Effects of SAP-released Tβ4 on proliferation and migration of EPDCs

**Figure 3:**
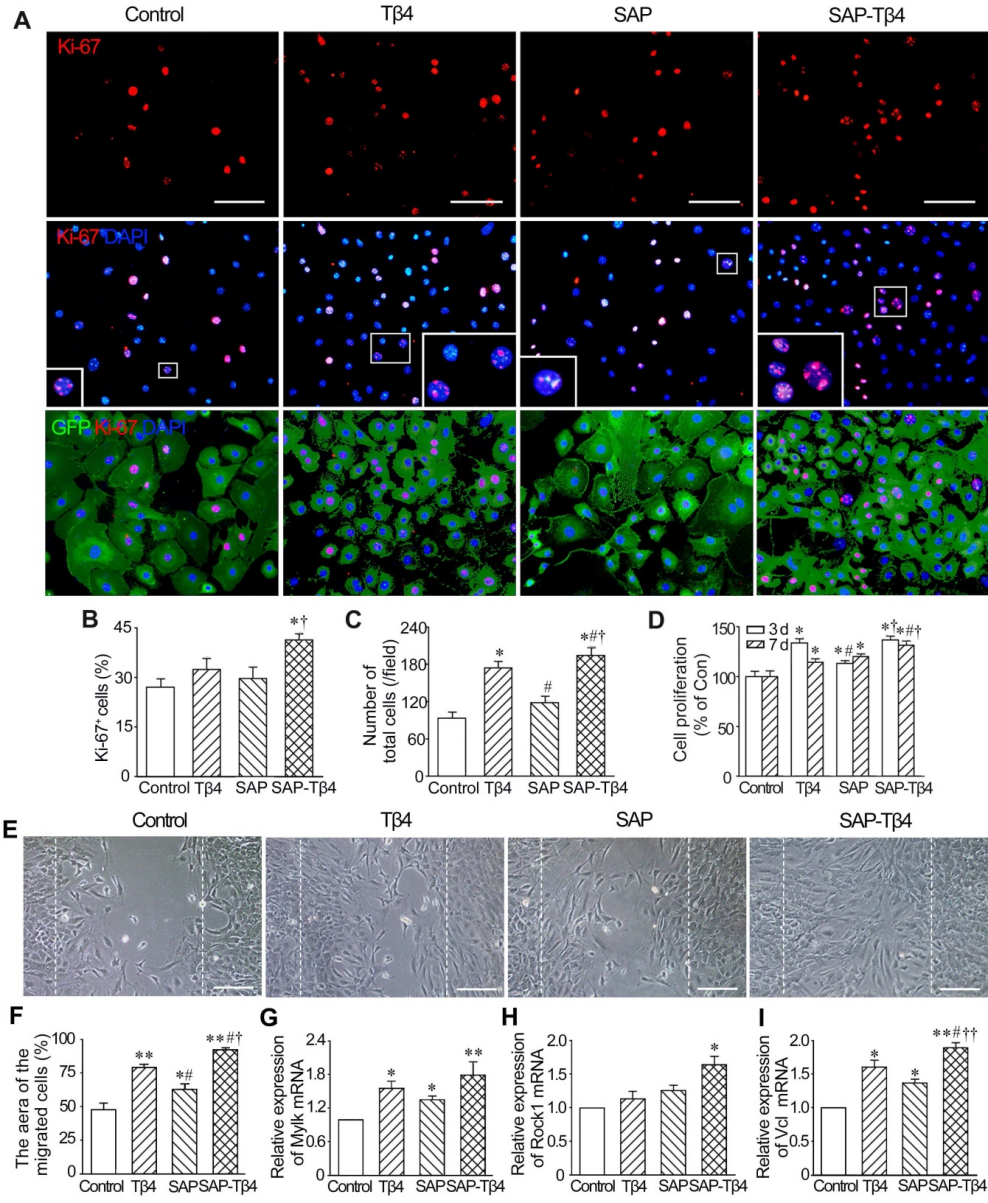
** SAP-released Tβ4 promotes proliferation and migration of EPDCs. (A)** The proliferated cells. The cells were treated for 3 days. The large boxes are magnification of the small boxes. Ki67 immunostaining. Scale bar = 100 µm. **(B)** Statistical result of the number of Ki67^+^ cells. **(C)** The number of total cells per field. n = 5. **(D)** Viability of the cells. The cells were treated for 3 days or 7 days. CCK-8 analysis. n = 5. *p < 0.05 versus control group; #*p* < 0.05 versus Tβ4 group; †*p* < 0.05 versus SAP group. **(E)** The cells migrated into the scratched region. The cells were incubated for 16 h after scratch. The dotted lines indicate the margin of the scratch. Scale bar = 100 µm. **(F)** Statistical result of the area of the migrated cells. n = 5. **(G-I)** Expression of *Mylk*,* Rock1* and *Vcl* at 16 h after scratch. qRT-PCR analysis. *p < 0.05 and **p < 0.01 versus control group; #*p* < 0.05 versus Tβ4 group; †*p* < 0.05 and ††*p* < 0.01 versus SAP group. n = 4.

The results of Ki67 immunostaining showed that the number of Ki67^+^ EPDCs in SAP-Tβ4 group was greater than that in control, Tβ4 and SAP groups. The number of total cells in the field in SAP-Tβ4 group was also increased significantly (Figure 3A-C). The results of CCK-8 analysis showed that cell proliferation occurred mostly by 3 days after treatment. Tβ4 and SAP promoted a modest degree of cell proliferation while proliferation was more prominent in SAP-Tβ4-treated cells than that of Tβ4 or SAP treatment alone (Figure 3D). To assess the migration of EPDCs due to Tβ4 released from SAP, we compared control, Tβ4 and SAP groups with the SAP-Tβ4 group and found that the area of the migrated cells was the greatest in the SAP-Tβ4-treated group. In addition, the area of the migrated cells in Tβ4 and SAP groups was greater than that in control group (Figure 3E and F). qRT-PCR analysis demonstrated that the level of expression of motility markers* Mylk*,* Rock1* and *Vcl* in SAP-Tβ4 group was significantly higher than that in control group. The expression of *Mylk* and *Vcl* in Tβ4 and SAP groups was also higher than that in control group. The expression of *Vcl* in SAP-Tβ4 group was higher than that of Tβ4 or SAP groups (Figure 3G-I).

### Effect of SAP-released Tβ4 on differentiation of EPDCs towards cardiovascular cells

**Figure 4:**
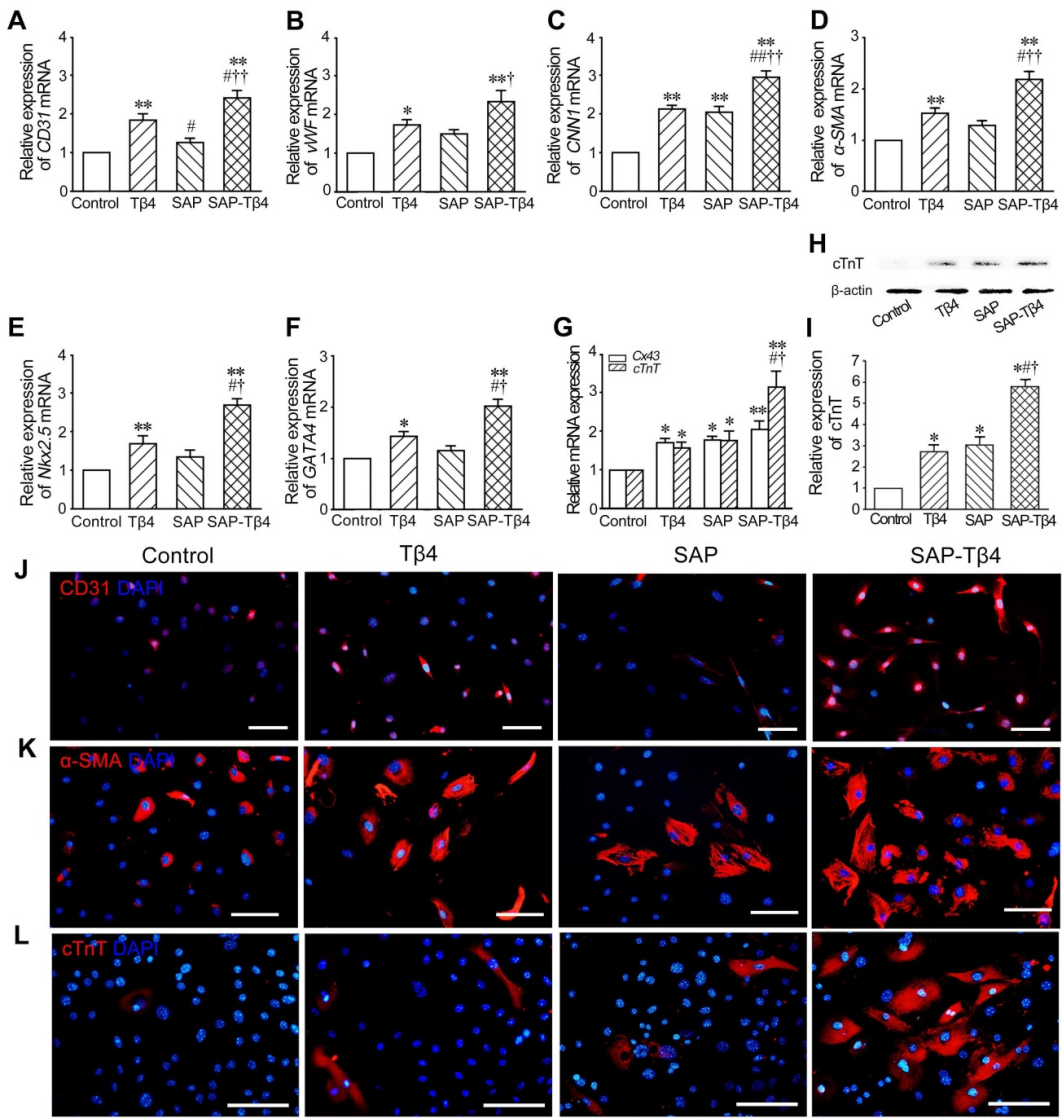
** SAP-released Tβ4 promotes differentiation of EPDCs towards cardiovascular cells. (A-G)** Expression of *CD31*, *vWF*, *CNN1*, *α-SMA*, *Nkx2.5, GATA4, Cx43* and* cTnT* in EPDCs after treatment. qRT-PCR analysis. *p < 0.05 and **p < 0.01 versus control group; #*p* < 0.05 and ##*p* < 0.01 versus Tβ4 group; †*p* < 0.05 and ††*p* < 0.01 versus SAP group. n = 4. **(H)** Expression of cTnT. Western blots. **(I)** Statistical result of Western blots. *p < 0.05 versus control group; #*p* < 0.05 versus Tβ4 group; †*p* < 0.05 versus SAP group. n = 3. **(J, K)** Expression of CD31 and α-SMA in the cells at 2 weeks after treatment. **(L)** Expression of cTnT in the cells at 4 weeks after treatment. Immunostaining. Scale bar = 50 µm.

The results of qRT-PCR showed that expression of *CD31*, *vWF*, *CNN1*, *α-SMA*, *Nkx2.5, GATA4, Cx43* and* cTnT* in the cells was increased in Tβ4 group. In SAP group, expression of *CNN1*, *Cx43* and* cTnT* was also increased. Compared with Tβ4 and SAP groups, expression of the genes in SAP-Tβ4 group was significantly higher (Figure 4A-G). The result of Western blots of cTnT expression (Figure 4H and I) was consistent with those from qRT-PCR. Immunostaining showed an increase in CD31^+^, α-SMA^+^ and cTnT^+^ cells in Tβ4-treated group that presumably differentiated from GFP^+^ EPDCs precursors. In SAP-Tβ4-treated group there were more cells expressing CD31, α-SMA or cTnT than those treated with Tβ4 alone (Figure 4J-L, Figure S3).

### Effect of SAP-released Tβ4 on paracrine activity of EPDCs

EPDCs have been described to secrete many cytokines/growth factors including bFGF, HGF, IGF-1, PDGF-BB, SCF, SDF-1, TGF-β1 and VEGF, among others. After treatment with Tβ4, the concentrations of bFGF, HGF, IGF-1, SDF-1, TGF-β1 and VEGF were increased. The concentration of paracrine factors in SAP-Tβ4 group was significantly higher than that in the Tβ4 group (Figure S4).

### Improvement of cardiac function and morphological changes of LV wall after implantation of SAP carrying Tβ4

**Figure 5:**
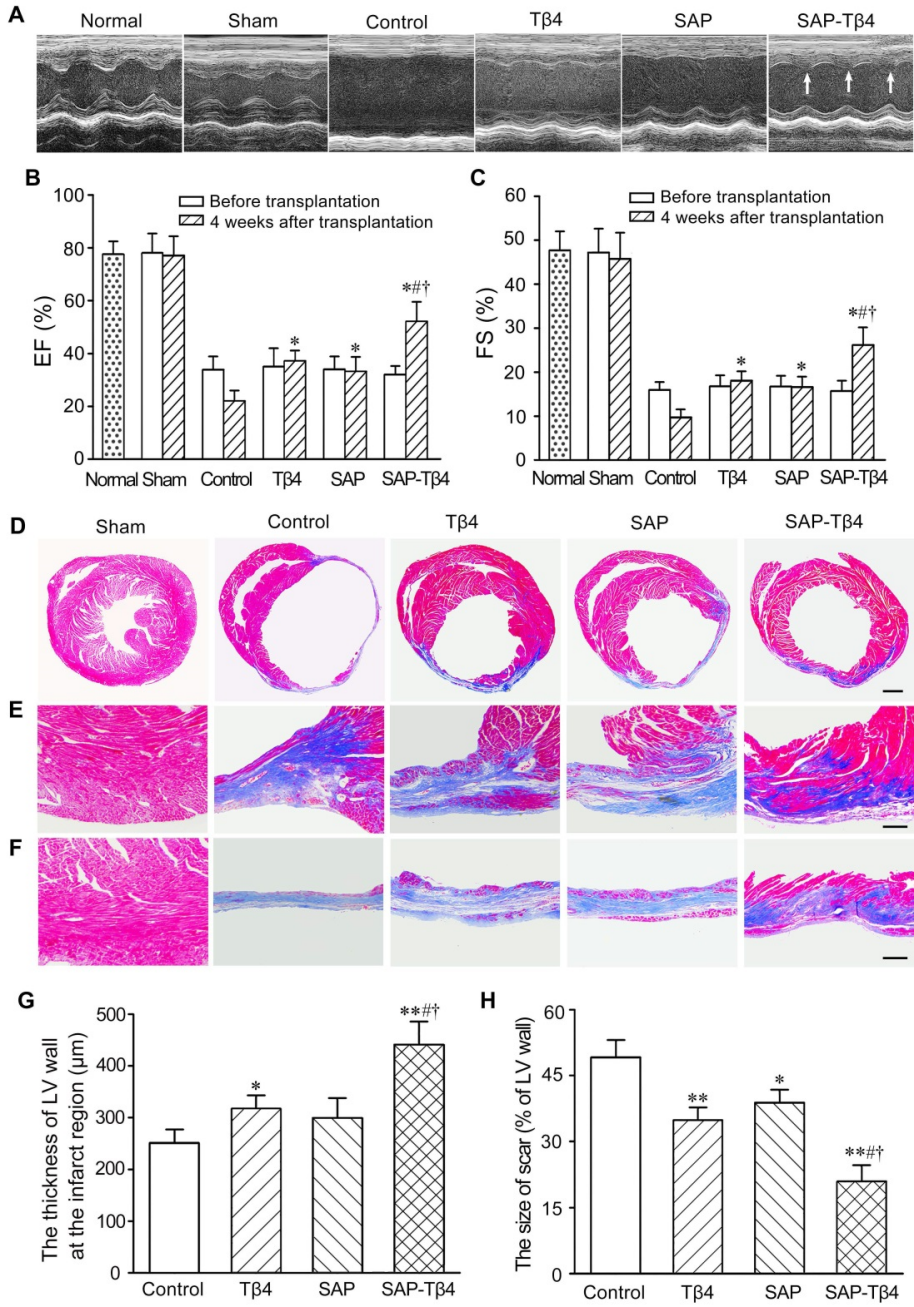
** The changes of function and structures of the hearts at 4 weeks after implantation. (A)** Representative echocardiograms of the LV free walls. Arrows indicate the contraction waves of LV anterior wall. **(B)** Ejection fractions (EF) of the left ventricle. **(C)** Fractional shortening (FS) of the left ventricle. EF and FS were assessed in normal mice, before implantation (1 week after MI) and at 4 weeks after implantation respectively. n ≧ 8. **(D-F)** The morphological changes of the walls of the left ventricle. The collagen-rich scar tissue was stained blue, while viable myocardial tissue was stained red. Panels in D show the transverse sections of the ventricles at the widest part of the infarct region. Panels in E and F show the transverse sections of the peri-infarcted and infarcted regions respectively. Masson's trichrome staining. Scale bar = 2 mm (D), 200 µm (E, F). **(G)** Statistical result of the thickness of LV wall at the infarct region. **(H)** Statistic result of scar size. *p < 0.05 and **p < 0.01 versus control group; #*p* < 0.05 versus Tβ4 group; †*p* < 0.05 versus SAP group. n ≧ 8

Representative echocardiograms of the LV free walls were shown in Figure 5A. The echocardiograms revealed that cardiac function in all mice was severely compromised at 1 week after MI. In the control group, the loss of cardiac function lasted for 4 weeks. At 4 weeks after implantation, LV contraction in Tβ4, SAP and SAP-Tβ4 groups was significantly improved. Compared with Tβ4 and SAP groups, LV contraction in SAP-Tβ4 group was strengthened dramatically. EF and FS of healthy mice were 78.45±4.13% and 48.43±5.41% respectively. After implantation, the EF and FS were increased in all groups, while the SAP-Tβ4 group showed a remarkably greater EF and FS than those in Tβ4 and SAP groups (Figure 5B and C).

The morphological changes of LV wall were shown in Figure 5D-F. In control group, the myocardium of the infarcted region was replaced by fibrous tissue at 4 weeks after implantation. There was more myocardium at the infarct region in Tβ4 and SAP-Tβ4 groups than that in control group. The thickness of the LV wall in SAP-Tβ4 group was greater than that in Tβ4 group. The scar size was decreased in Tβ4, SAP, and SAP-Tβ4 groups. The scar size in the SAP-Tβ4 group was significantly smaller than those in the Tβ4 and SAP groups (Figure 5G and H). Interestingly, the SAP is degraded completely at one month after implantation.

### Activation of the epicardium after implantation

**Figure 6:**
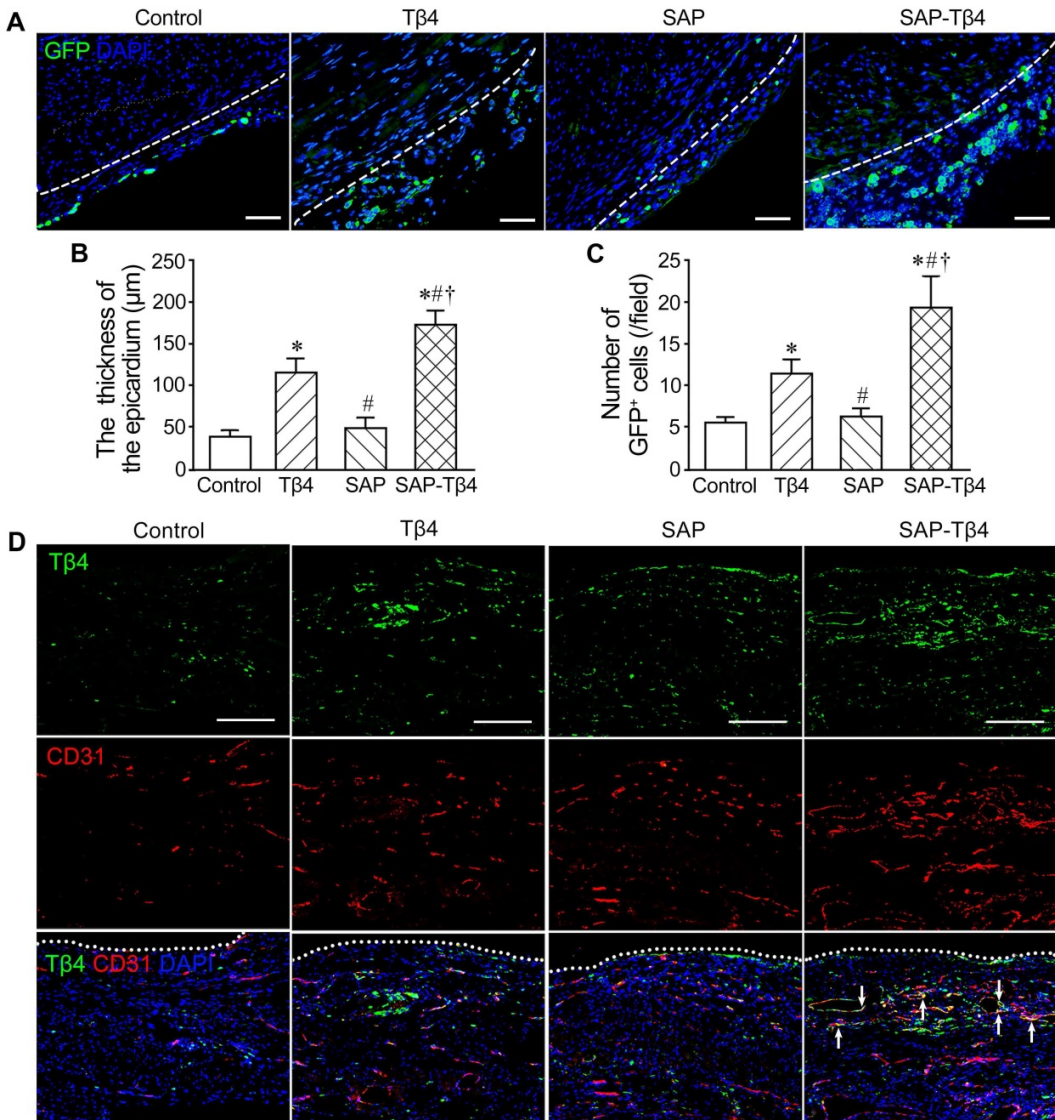
**The activated epicardium and Tβ4^+^ cells in the epicardium and myocardium at 1 week after implantation. (A)** The epicardium and GFP^+^ EPDCs. Immunostaining. The dotted lines indicate the junction between the epicardium and myocardium. Scale bar = 50 µm. **(B)** Statistical result of the thickness of the epicardium. **(C)** Statistical result of the number of EPDCs in the epicardium. *p < 0.05 versus control group; #*p* < 0.05 versus Tβ4 group; †*p* < 0.05 versus SAP group. n ≧ 5. **(D)** Tβ4 expression in the epicardium and myocardium. Immunostaining. Tβ4 is mainly expressed in the vascular endothelial cells (arrows) near the epicardium. The dotted lines indicate the surface of the epicardium. Scale bar = 200 µm.

The activated EPDCs are known to express WT1. WT1 expression is turned off when EPDCs are fully differentiated. EPDCs in the epicardium were barely detectable in sham group. After MI, the epicardium at peri-infarcted region became thickened. At 1 week after MI, EPDCs in the thickened epicardium at peri-infarcted region were detectable. After Tβ4 injection, the epicardium became thicker and the number of GFP^+^ cells in the epicardium were increased. After implantation of SAP carrying Tβ4, the epicardium was thickened even further and the number of GFP^+^ cells was greater (Figure 6A-C).

### Increase of Tβ4^+^ cells in the epicardium and myocardium after implantation

The results of immunostaining demonstrated that Tβ4 was expressed in the epicardium and myocardium at the peri-infarct region at 1 week after MI. There were more Tβ4^+^ cells in the Tβ4 group than control group. Compared with Tβ4 group, Tβ4^+^ cells in SAP-Tβ4 group were increased. Double staining Tβ4 and CD31 showed that Tβ4 was mainly expressed in the endothelial cells of the microvessels in the myocardium near the epicardium (Figure 6D).

### Migration and differentiation of EPDCs into cardiovascular and lymphatic cells after implantation

**Figure 7:**
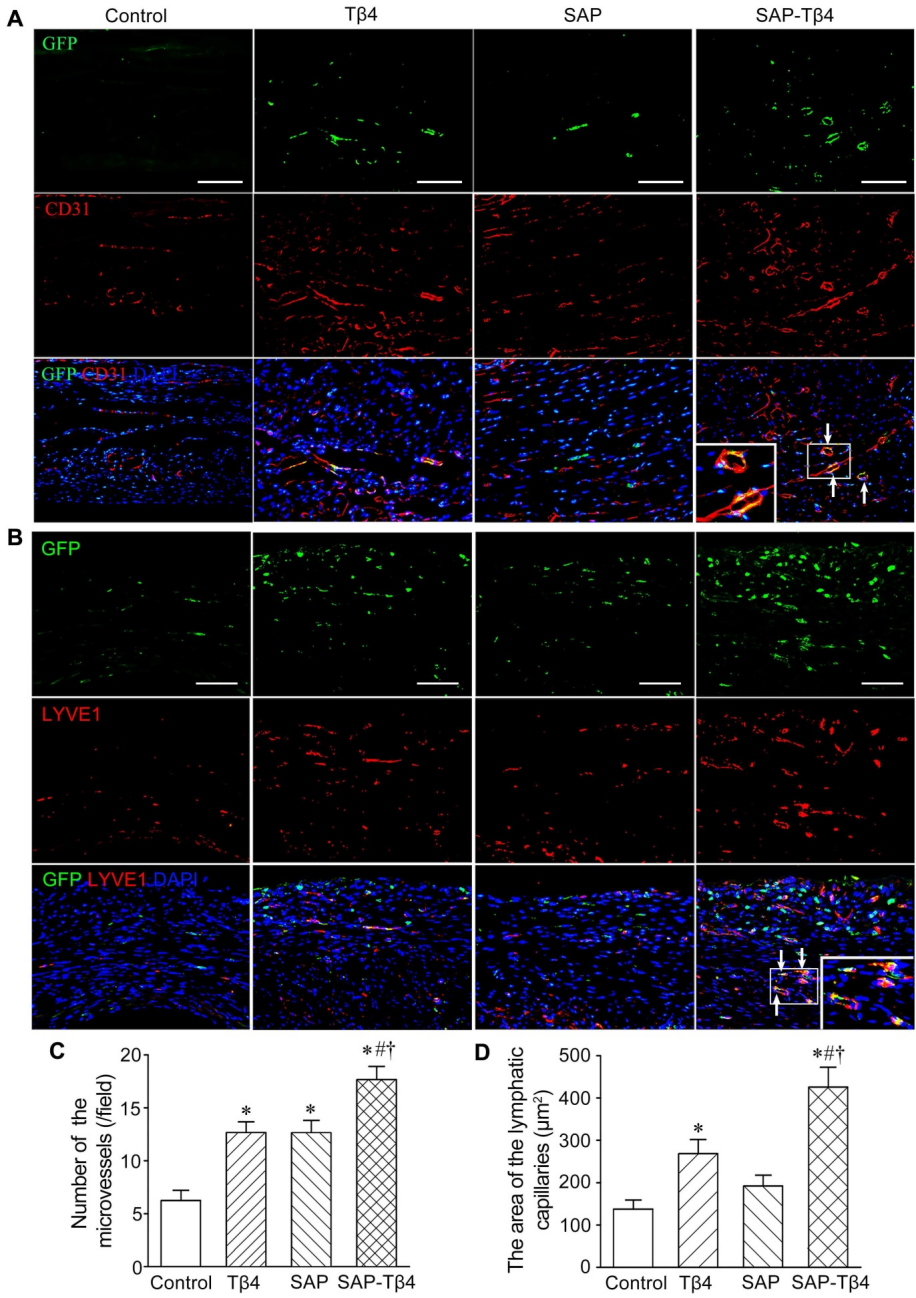
** EPDCs differentiate into vascular and lymphatic endothelial cells at 4 weeks after implantation. (A)** CD31^+^ cells differentiated from EPDCs at the infarct region. The differentiated cells (arrows) are located at the wall of the microvessels. **(B)** LYVE-1^+^ cells differentiated from EPDCs at peri-infarct region. The differentiated cells from EPDCs (arrows) are located at the wall of the lymphatic vessels. Immunostaining. The large boxes are magnification of the small boxes. Scale bar = 100 µm. **(C)** Statistical result of the density of the microvessels. *p < 0.01 versus control group; #*p* < 0.01 versus Tβ4 group; †*p* < 0.01 versus SAP group. n ≧ 5. **(D)** Statistical result of the area of the lymphatic capillaries. *p < 0.01 versus control group; #*p* < 0.05 versus Tβ4 group; †*p* < 0.01 versus SAP group. n ≧ 5.

**Figure 8:**
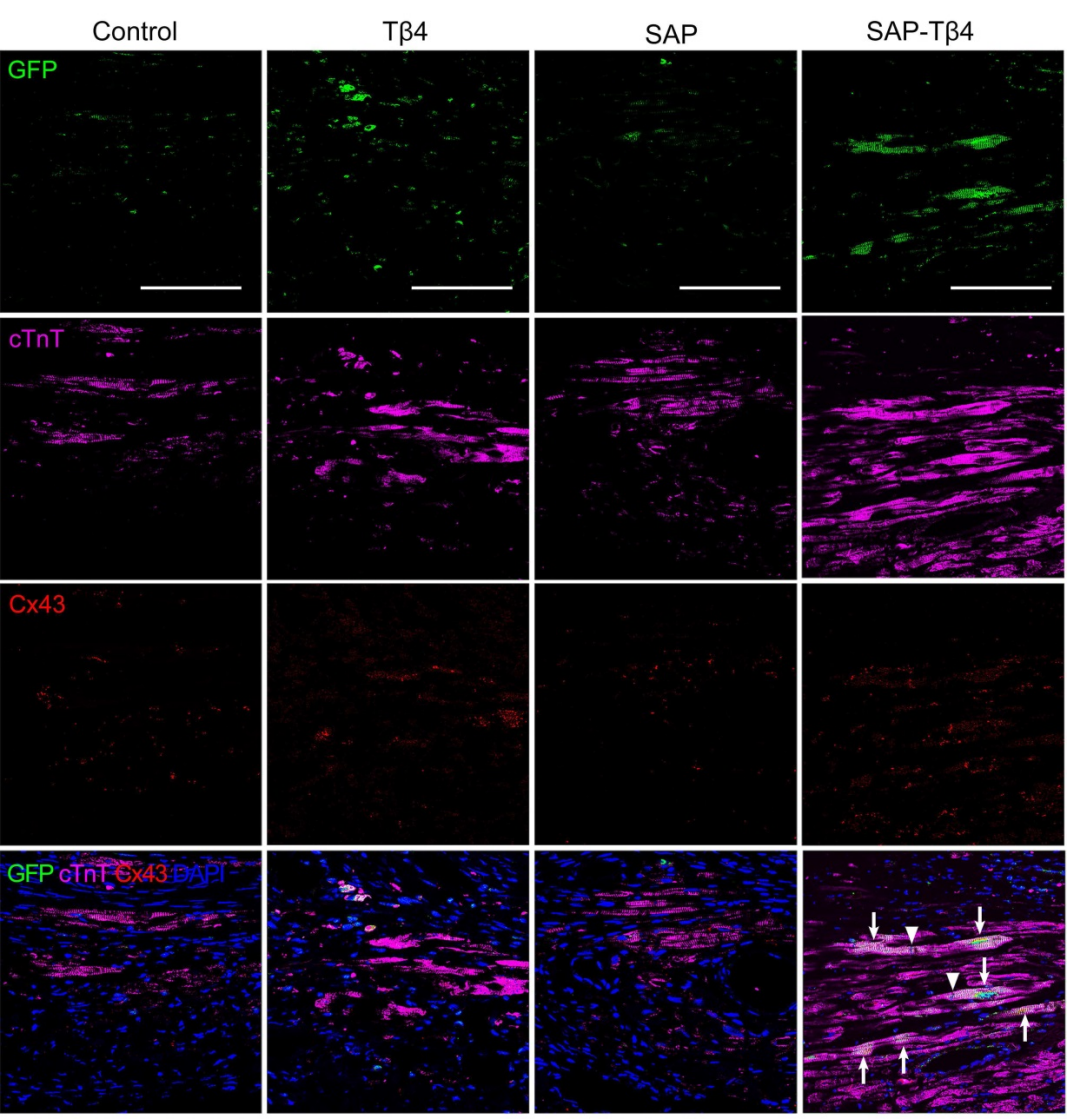
** EPDCs differentiate into cardiomyocytes at 4 weeks after implantation.** Arrows indicate GFP^+^ and cTnT^+^ double positive cells differentiated from EPDCs. Triangles indicate Cx43 expression at the junction between cardiomyocyte differentiated from EPDC and other *de novo* cardiomyocyte. Scale bar = 50 µm.

At 4 weeks after implantation, EPDCs migrated into the myocardium, and the epicardium became thinner. EPDCs were distributed beneath the epicardium and in the myocardium. The migration of cells into the myocardium was more in the SAP-Tβ4 group than in the Tβ4 and SAP groups. In the Tβ4 group, a few EPDCs expressed CD31, LYVE-1, α-SMA or cTnT. The SAP-Tβ4 group showed more EPDCs expressing CD31, LYVE-1, α-SMA or cTnT than the Tβ4 group (Figure 7, Figure S5, Figure 8). EPDC-derived CD31^+^ endothelial cells or a-SMA^+^ smooth muscle cells were found to incorporate into the wall of the microvessels, while EPDC-derived LYVE-1^+^ endothelial cells were incorporated to the wall of the lymphatic capillaries (Figure 7, Figure S5). EDPC-derived GFP^+^cTnT^+^ cardiomyocytes were found adjacent to native cardiomyocytes (Figure 8).

### Angiogenesis, lymphangiogenesis and myocardial regeneration after implantation

Angiogenesis at the infarct region and lymphangiogenesis at peri-infarct region were assessed by counting the number of CD31^+^ microvessels and measuring the area of LYVE-1^+^ lymphatic vessels. In Tβ4, SAP, and SAP-Tβ4-treated groups, the density of the microvessels at the infarct region was increased significantly at 4 weeks after implantation compared with control. In comparison with the Tβ4 and SAP groups, the density of the microvessels in the SAP-Tβ4 group was significantly greater (Figure 7C). The area of the lymphatic vessels at the peri-infarct region was increased significantly in both Tβ4 and SAP-Tβ4-treated groups, with a greater increase in the SAP-Tβ4 group compared with the Tβ4 group (Figure 7D). In addition to differentiation of EPDCs into cardiomyocytes, myocardial regeneration in the infarct region was evaluated by triple-labelled immunostaining of GFP, cTnT and Cx43. In the control group, there was less myocardium at the infarct region at 4 weeks after implantation, and Cx43 expression was not observed. There was more GFP^+^cTnT^+^ and Cx43^+^ cardiomyocytes in Tβ4, SAP and SAP-Tβ4-treated groups, especially in the SAP-Tβ4 group. Cx43 expression was noted at the junction between endogenous cTnT^+^ cardiomyocytes and *de novo* cardiomyocytes differentiated from EPDCs (Figure 8).

**Figure 9:**
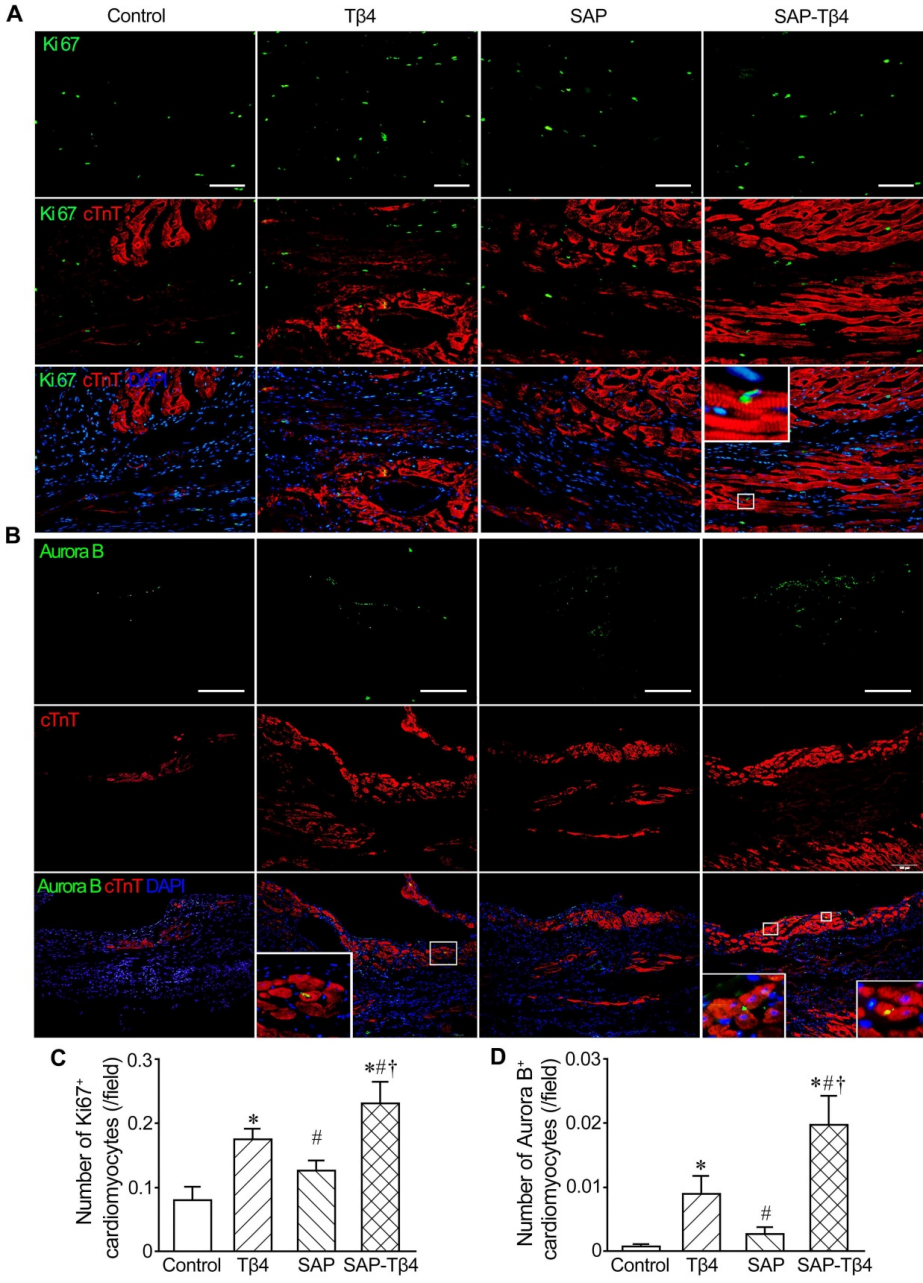
** Cardiomyocyte proliferation at 4 weeks after implantation. (A)** Ki67^+^ cardiomyocytes. **(B)** Aurora B^+^ cardiomyocytes. Immunostaining. The large boxes are magnification of the small boxes. (**C**) Statistical result of the number of Ki67^+^ cardiomyocytes. (**D**) Statistical result of the number of Aurora B^+^ cardiomyocytes. Scale bar = 100 µm. *p < 0.05 versus control group; #*p* < 0.05 versus Tβ4 group; †*p* < 0.05 versus SAP group.

We also assess the proliferation of cardiomyocytes with double immunostaining of cTnT and Ki67 or Aurora B. In the control and SAP groups, few cardiomyocytes expressed Ki67, while the expression of Aurora B was not observed in control group. In the Tβ4-treated group there were more Ki67^+^ and Aurora B^+^ cardiomyocytes. In the SAP-Tβ4-treated group, the frequency of Ki67^+^ or Aurora B^+^ cardiomyocytes were significantly increased (Figure 9).

### Enhancement of paracrine in the infarcted myocardium after implantation

Expression of *bFGF, HGF, HIF-1α, IGF-1, PDGF-BB, SCF, SDF-1 and VEGF* in Tβ4 group was increased at 1 week after implantation. In SAP-treated group, the expression of *bFGF*, *PDGF-BB*, *SDF-1* and *VEGF* were increased. Compared with Tβ4- and SAP-treated groups, the expression of genes in SAP-Tβ4-treated group was significantly higher (Figure S6).

## Discussion

In this study, we examine whether activating the epicardium with sustained Tβ4 release from a functionalized SAP could be a potential therapy for the repair of the infarcted myocardium. The functionalized SAP consists of a designer RADA-RPR that contains a self-assembling motif, a Tβ4-binding site, and a cell adhesive ligand. The adhesive ligand on the SAP likely binds to integrins expressed on the cell surface. The binding and encapsulation of Tβ4 within the SAP may prevent its rapid degradation and dissipation into the bloodstream. Following the degradation of SAP, the SAP-carried Tβ4 is then slowly released 33. Compared with RADA16-I which is one of traditional SAP, Tβ4 release from RADA-RPR is sustained over a longer period of time. Furthermore, the RGDS motif of RADA-RPR may promote homing and growth of EPDCs and other cells. In addition, the SAP demonstrates good biocompatibility, safety, and biodegradation properties that mimics natural cardiac extracellular matrix 34. Of note, the administration of cardiac extracellular matrix has been shown to facilitate epicardium-mediated heart regeneration 35. Intramyocardial injection of SAP reduces adverse ventricular remodeling 36. SAP hydrogel creates extracellular matrix-like microenvironment in the myocardium and promotes recruitment of vascular cells and vascularization 37.

In this study, we found a favorable biocompatibility of RADA-RPR with the tissue without immunogenicity. The microenvironment created by RADA-RPR appears to be suitable for survival and differentiation of EPDCs. With regards to the activation of the epicardium, the local sustained release with functionalized SAP may be more effective in delivering a higher and more durable concentration of Tβ4 than intraperitoneal injection or intravenous infusion. Recent study has suggested that epicardial microneedle patch allows regenerative factors to be released into the injured myocardium 38. In view of having dual functions of binding Tβ4 and adhering to epicardial cells, the RADA-RPR SAP may also be useful for delivering Tβ4 and EPDCs or stem cells simultaneously for optimizing MI therapy.

The results of this study demonstrate that local sustained release of Tβ4 with the functionalized SAP may effectively activate the epicardium. SAP-released Tβ4 can induce EPDCs to proliferate, migrate into subepicardium and myocardium, and differentiate towards the cardiovascular cells. At 4 weeks after SAP-Tβ4 delivery, enhanced neovascularization and myocardial regeneration were noted, adverse myocardial remodeling was attenuated, and cardiac function improved significantly. While priming with Tβ4 followed by MI may augment epicardial response in myocardial regeneration 15, 23, it has suggested that differentiation of EPDCs into cardiomyocytes in the injured adult heart is controversial 39, limiting its application as a MI therapy. Tβ4 treatment presents augmented angiogenesis and reduced infarct size 25-27, while effectiveness of Tβ4 treatment on EPDC differentiation has not been evaluated. In our experiments, WTl tracing confirms that EPDCs can differentiate into endothelial cells and smooth muscle cells as well as cardiomyocytes after induction with SAP-released Tβ4. The junctions of EPDC-differentiated cardiomyocytes and other *de novo* cardiomyocytes express Cx43, which indicates that the regenerated myocardium is electrically connected with the native myocardium. Since there are fewer EDPC-differentiated cardiomyocytes in the infarcted myocardium with Tβ4 injection only, the delivery of Tβ4 with functionalized SAP should extend the application of Tβ4 treatment in repair of the infarcted myocardium.

Besides activating the epicardium, Tβ4 has cytoprotective 17, 26, 40, 41 and anti-inflammatory effects 26. In this experiment, the effect of Tβ4 released from SAP on EPDC survival was greater than that of Tβ4 only. On the other hand, EPDCs participate in repair of the infarcted myocardium via paracrine mechanism 42. Our experimental data show that SAP-released Tβ4 promotes paracrine of EPDCs. Moreover, the level of Tβ4 in the epicardium and myocardium is increased after delivery of Tβ4 with the SAP. Thus, endogenous Tβ4 may also contribute to the activation of the epicardium and differentiation of EPDCs. In addition, the results of this study demonstrate that proliferation of cardiomyocytes contributes to Tβ4-induced myocardial regeneration.

In this study we show for the first time that EPDCs have a potential to differentiate towards lymphatic endothelial cells when stimulated by SAP-released Tβ4. Moreover, lymphangiogenesis at the peri-infarct region is enhanced after delivery of SAP-Tβ4. Our observations suggest that the processes of EPDC-induced lymphangiogenesis include migration of EPDCs into subepicardium and myocardium, differentiation into lymphatic endothelial cells, incorporation to the wall of the lymphatic capillary, and participation in lymphangiogenesis. The cardiac lymphatic vessels drain excessive protein and fluid from the extracellular spaces and transport leukocytes and antigen-presenting cells from the inflammatory tissue. Impairment of lymphatic drainage may result in cardiac edema, inflammation, and fibrosis post-MI 43. Although an increase in the number of the lymphatic vessels in the peri-infarcted region is a positive response post-MI 44, 45, these new-formed lymphatic vessels are insufficient for cardiac lymphatic drainage. Therefore, promoting lymphangiogenesis is considered as a potential strategy for resolution of insufficient cardiac lymphatic drainage. Our recent study suggests that the delivery of lymphatic endothelial progenitor cells and VEGF-C with SAP promotes cardiac lymphangiogenesis and effectively repairs the infarcted myocardium 46. Moreover, transplantation of mesenchymal stem cell-loaded cardiac patch on the epicardium with MI enhances cardiac lymphangiogenesis 30. However, the contribution of the epicardium to cardiac lymphangiogenesis post-MI is unknown. The results of this study demonstrate that differentiation of EPDCs towards lymphatic endothelial cells and paracrine of growth factors from EPDCs account for enhancement of lymphangiogenesis. In light of the potential of EPDCs to differentiate towards lymphatic endothelial cells, the epicardium may represent an ideal therapeutic target to trigger cardiac lymphangiogenesis post-MI.

## Conclusion

We found in this study that the designer RADA-RPR bound to Tβ4 can provide a sustained release of Tβ4 *in vivo* and adhere to EPDCs. The intracardiac delivery of SAP-Tβ4 activates the epicardium, enhances myocardial regeneration, and improves cardiac function post-MI. SAP-released Tβ4 promotes differentiation of EPDCs towards cardiovascular cells and lymphatic endothelial cells. Moreover, SAP-released Tβ4 has cytoprotective effect on the survival of EPDCs and augments paracrine of the cells. We propose that the intracardiac delivery of SAP-Tβ4 represents a novel approach to activate the epicardium and enhance the repair of the infarcted myocardium. Local delivery of SAP-Tβ4 in large animal models such as pigs may achieved by catheter-based transendocardial injection or intraventricular injection guided by echocardiography for future translational studies.

## Supplementary Material

Supplementary figures and tables.

## Abbreviations

AFM: atomic force microscope
α-SMA: α-smooth muscle actin
bFGF: basic fibroblast growth factor
CCK-8: cell counting kit-8
cTnT: cardiac troponin T
Cx43: connexin-43
DAPI: 4', 6-diamidino-2-phenylindole
DMEM: Dulbecco's modified Eagle's medium
EB/AO: ethidium bromide/acridine orange
ELISA: enzyme-linked immunosorbent assay
EMT: epithelial to mesenthymal transition
EPDCs: epicardium-derived cells
FACS: fluorescence-activated cell sorter
FBS: fetal bovine serum
GFP: green fluorescent protein
HGF: hepatocyte growth factor
HIF-1α: hypoxia-inducible factor 1-α
IGF-1: insulin-like growth factor 1
LAD: left anterior descending coronary artery
LDH: lactate dehydrogenase
LVEDD: left ventricular end-diastolic internal diameter
LVEDV: left ventricular end-diastolic volume
LVESD: left ventricular end-systolic internal diameter
LVESV: left ventricular end-systolic volume
LYVE-1: lymphatic vessel endothelial hyaluronan receptor 1
MI: myocardial infarction
Mylk: myosin light chain kinase
OCT: optimal cutting temperature compound
PDGF-BB: platelet-derived growth factor BB
qRT-PCR: quantitative reverse transcription-polymerase chain reaction
Rock1: Rho-associated protein kinase 1
SAP: self-assembling peptide
SCF: stem cell factor
SDF-1: stromal cell-derived factor 1
SEM: scanning electron microscope
Tβ4: thymosin β4
Tbx18: T-box factor 18
TGF-β1: transforming growth factor β1
UPLC-MS/MS: ultra-performance liquid chromatography-tandem mass spectrometry
Vcl: vinculin
VEGF: vascular endothelial growth factor
vWF: von Willebrand factor
WT1: Wilms tumor 1
