# Supplementary material for: Thymosin β4 released from functionalized self-assembling peptide activates epicardium and enhances repair of infarcted myocardium
Source: Theranostics. 2021 Feb 20;11(9):4262–80. doi: 10.7150/thno.52309 (PMC7977468; doi:10.7150/thno.52309)
Supplement: Supplementary file 1 — Supplementary figures and tables. [file thnov11p4262s1.pdf]

SUPPLEMENTARY MATERIAL

Thymosin β4 released with functionalized self-assembling peptide activates epicardium and enhances repair of infarcted myocardium

Running head: Activating Epicardium by SAP-released Tβ4

Yong-li Wang[1,2], Shu-na Yu[1], Hao-ran Shen[1], Hai-jie Wang[1,*], Xue-ping Wu[1], Qiang-li Wang[1,3], Bin Zhou[4], Yu-zhen Tan[1,*]

1. Department of Anatomy, Histology and Embryology, Shanghai Medical School of Fudan University, Shanghai 200032, China
2. Laboratory of Oral Microbiota and Systemic Diseases, Shanghai Ninth People's Hospital, College of Stomatology, Shanghai Jiao Tong University School of Medicine, Shanghai 200125, China.
3. School of Basic Medical Sciences, Shanghai University of Traditional Chinese Medicine, Shanghai 201203, China.
4. Shanghai Institute of Biochemistry and Cell Biology, Chinese Academy of Sciences, Shanghai 200032, China

To whom correspondence should be addressed to:

Yu-zhen Tan Professor, MD., PhD.

Department of Anatomy, Histology and Embryology

Shanghai Medical School of Fudan University

138 Yixueyuan Road, Shanghai 200032, People's Republic of China

Tel: +86-21-54237019-9306

E-mail: yztan@shmu.edu.cn

* Corresponding author: Department of Anatomy, Histology and Embryology, Shanghai Medical School of Fudan University, 138 Yixueyuan Road, Shanghai 200032, People's Republic of China.

E-mail: yztan@shmu.edu.cn (YZ Tan); hjwang@shmu.edu.cn (HJ Wang)

Table S1. The sequences of the primers

Symbol	Version	Sequence (5' to 3')	Length
bFGF	NM_008006.2	(F) GGCTGCTGGCTTCTAAGTG	101 bp
		(R) CCAACTGGAGTATTTCCGTGA	
CD31	NM_001032378.2	(F) GCCAAGGCCAAACAGAAACC	178 bp
		(R) CCATGTTCTGGGGGTCTTTAT	
CNN1	NM_009922.4	(F) CCCACAATCACCACCCGCACAACTA	165 bp
		(R) TCATCTCCCCAAACCGTAACCCTAT	
cTnT	NM_001130174.2	(F) CGGGCGTTGGAAATAGATGA	165 bp
		(R) TAGGGGTCAGGCAGAGTACT	
Cx43	NM_010288.3	(F) AAGGCGTGAGGGAAGTACCA	215 bp
		(R) GGAGTAGGCTTGGACCTTGT	
GATA4	NM_001310610.1	(F) GGGATTCAAACCAGAAAACG	198 bp
		(R) GCTGTGCCCATAGTGAGATG	
HGF	NM_001289458.1	(F) GGGACGGTATCCATCACT	186 bp
		(R) TTCGTAGCGTACCTCTGG	
HIF-1α	NM_001313919.1	(F) TCGGCGAAGCAAAGAGTC	182 bp
		(R) CCATCTGTGCCTTCATCTCA	
IGF-1	NM_001111274.1	(F) TGCCACATCACCGCAGGAT	200 bp
		(R) CCACGCCAGGACCACTTT	
Mylk	NM_139300.3	(F) AAAAACCGTCTGGACTGCAC	145 bp
		(R) TCACAGCATTGCCCGTTTTC	
Nkx2.5	NM_008700.2	(F) CAGTGGAGCTGGACAAAGCC	217 bp
		(R) TAGCGACGGTTCTGGAACCA	
PDGF-BB	NM_011057.3	(F) TTAGCGGGCGAGTGAAGACG	196 bp
		(R) GGGAGGACCTGGACAAGGGA	
Rock1	NM_009071.2	(F) AAGCTTTTGTTGGCAATCAGC	129 bp
		(R) AACTTTCCTGCAAGCTTTTATCCA	
SCF	NM_001347156.1	(F) GTCATTGTTGGCTACGAG	145 bp
		(R) CATAACACGAGGTCATCC	
SDF-1	NM_001012477.2	(F) GTCAGCCTGAGCTACCGA	103 bp
		(R) GAAGGGCACAGTTTGGAG	
Tbx18	NM_023814.4	(F) GTGGAGTCATACGCATTCTGGA	141 bp
		(R) GTGAGGATGTGTAGCAGGGACA	
Tcf21	NM_011545.1	(F) CATTCACCCAGTCAACCTGA	71 bp
		(R) CCACTTCCTTCAGGTCATTCTC	
Vcl	NM_009502.4	(F) AACCAGCCAATGATGATGGC	89 bp
		(R) TTGGCTGCTGCAATGATGTC	
VEGF	NM_001025250.3	(F) ACTATTCAGCGGACTCACCAG	171 bp
		(R) TGAGGGAGTGAAGAACCAACC	
vWF	NM_011708.4	(F) GCGGTGTAAACGGACATCTC	232 bp
		(R) ACAGGTTCGGGCATACTCAA	
WT1	NM_144783.2	(F) GCCTTCACCTTGCACTTCTC	186 bp

		(R) GACCGTGCTGTATCCTTGGT	
α-SMA	NM_007392.3	(F) GGCTCTGGGCTCTGTAAGG	149 bp
		(R) CTCTTGCTCTGGGCTTCATC	
β-actin	NM_007393.5	(F) GGAGATTACTGCTCTGGCTCCTA	150 bp
		(R) GACTCATCGTACTCCTGCTTGCTG	

Table S2. The primary sequences of the self-assembling peptides

Code	Primary sequence
RADA16-I	Ac-RADARADARADARADA-$CONH_2$
RADA-RPR	Ac-$(RADA)_4$GGRPRHQGVMRGDS-$CONH_2$

Table S3. The antibodies used

Antibody	Dilution	Company	Application
Chicken anti-GFP	1:100	Novus Biologics, Littleton, CO, USA	Immunofluorescence
Rabbit anti-Aurora B	1:100	Sigma-Aldrich,Saint Louise, MO, USA	Immunofluorescence
Rabbit anti-CCR7	1:100	Abcam, Cambridge, MA, USA	Immunofluorescence
Rabbit anti-Cx43	1:100	Abcam, Cambridge, MA, USA	Immunofluorescence
Rabbit anti-GFP	1:50	Santa Cruz Biotech, Dallas, TX,USA	Immunofluorescence
Rabbit anti-LYVE-1	1:200	Novus Biologics, Littleton, CO, USA	Immunofluorescence
Rabbit anti-Tbx18	1:100	Abcam, Cambridge, MA, USA	Immunofluorescence
Rabbit anti-Tβ4	1:200	Santa Cruz Biotech, CA, USA	Immunofluorescence
Rabbit anti-WT1	1:100	Santa Cruz Biotech, CA, USA	Immunofluorescence
Mouse anti-CD31	1:100	Abcam, Cambridge, MA, USA	Immunofluorescence
Mouse anti-CD31	1:100	BDBiosciences, San Jose, CA, USA	Immunofluorescence
Mouse anti-CD68	1:100	Santa Cruz Biotech, Dallas, TX,USA	Immunofluorescence
Mouse anti-cTnT	1:200	Santa Cruz Biotech, CA, USA	Immunofluorescence
Mouse anti-Ki-67	1:200	Abcam, Cambridge,	Immunofluorescence

		MA, USA	
Mouse anti-α-SMA	1:100	Abcam, Cambridge, MA, USA	Immunofluorescence
Goat anti-chicken (conjugated with DyLight 488)	1:400	Novus Biologics, Littleton, CO, USA	Immunofluorescence
Goat anti-mouse (conjugated with Alexa Fluor 594)	1:400	Jackson, West Grove, PA, USA	Immunofluorescence
Goat anti-mouse (conjugated with Alexa Fluor 488)	1:400	Jackson, West Grove, PA, USA	Immunofluorescence
Goat anti-mouse (conjugated with Alexa Fluor 647)	1:400	Jackson, West Grove, PA, USA	Immunofluorescence
Goat anti-rabbit (conjugated with Alexa Fluor 488)	1:400	Jackson, West Grove, PA, USA	Immunofluorescence
Goat anti-rabbit (conjugated with DyLight 594)	1:400	Abcam, Cambridge, MA, USA	Immunofluorescence
Mouse anti-cTnT	1:1000	Santa Cruz Biotech, CA, USA	Western Blotting
Mouse anti-β-actin	1:4000	Sigma-Aldrich, Saint Louise, MO, USA	Western Blotting

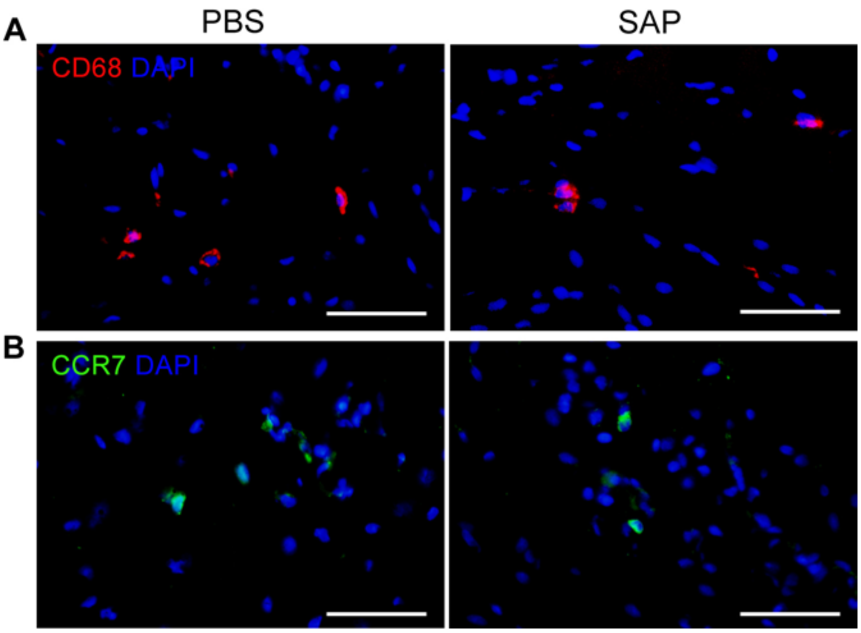

Figure S1. The distribution of CD68$^+$ or CCR7$^+$ immune cells at 1 week after

subcutaneous injection of the SAP. Immunostaining. Scale bar = 50 μm.

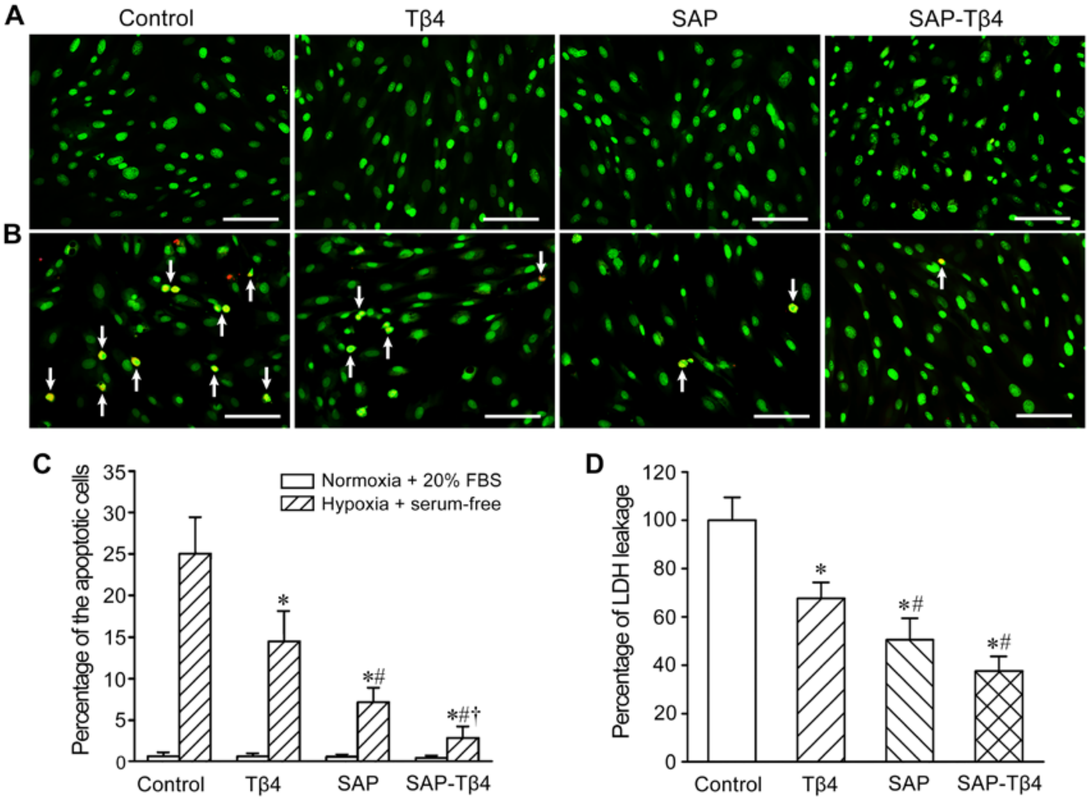

Figure S2. The protection of Tβ4 on survival of EPDCs. (A) EPDCs incubated in normoxic condition. **(B)** EPDCs treated with hypoxia (1% O_2) and serum-free for 12 h. Arrows indicate the apoptotic cells. EB/AO staining. Scale bar = 100 μm. **(C)** Statistical result of the number of the apoptotic cells. n = 5. **(D)** The concentration of LDH in the condition of hypoxia and serum deprivation. n = 5. *p < 0.01 versus control group; #p < 0.05 versus Tβ4 group; †p < 0.05 versus SAP group.

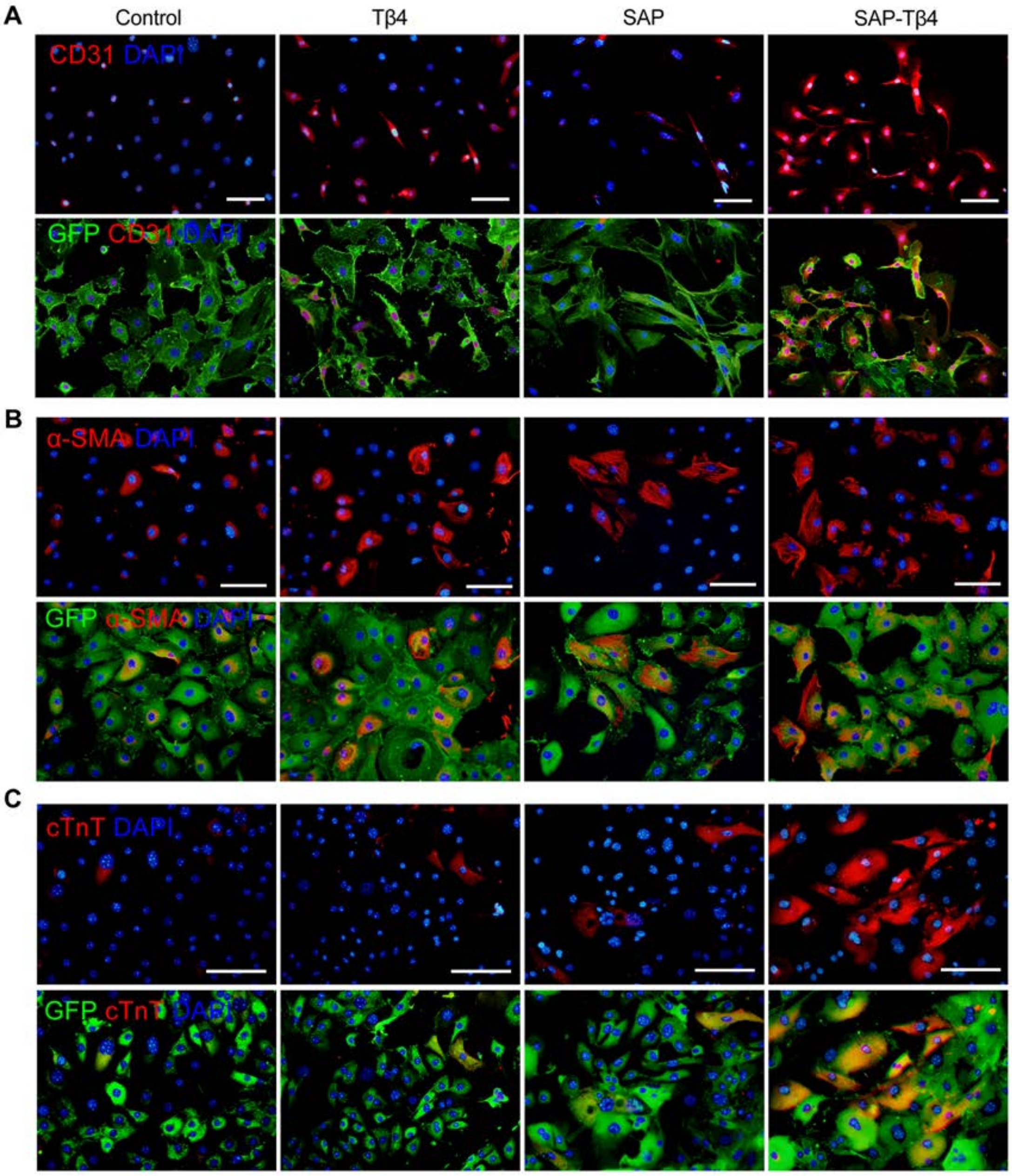

Figure S3. SAP-released Tβ4 promotes differentiation of EPDCs towards cardiovascular cells. (A) CD31^{+} cells differentiated from EPDCs. **(B)** α-SMA^{+} cells differentiated from EPDCs. **(C)** cTnT^{+} cells differentiated from EPDCs. Immunostaining. Scale bar = 50 μm.

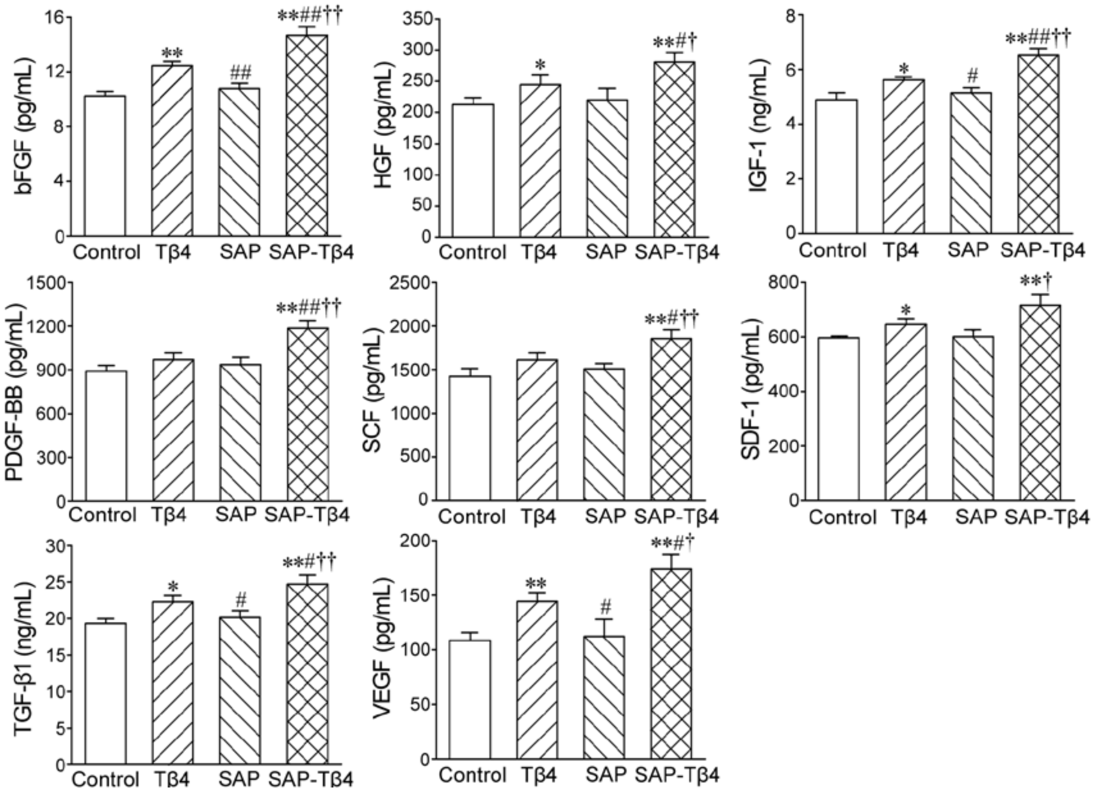

Figure S4. Paracrine factors secreted by EPDCs. The cells were incubated for one week. ELISA. **p* < 0.05 and ***p* < 0.01 versus control group; #*p* < 0.05 and ##*p* < 0.01 versus Tβ4 group; †*p* < 0.05 and ††*p* < 0.01 versus SAP group. n = 3.

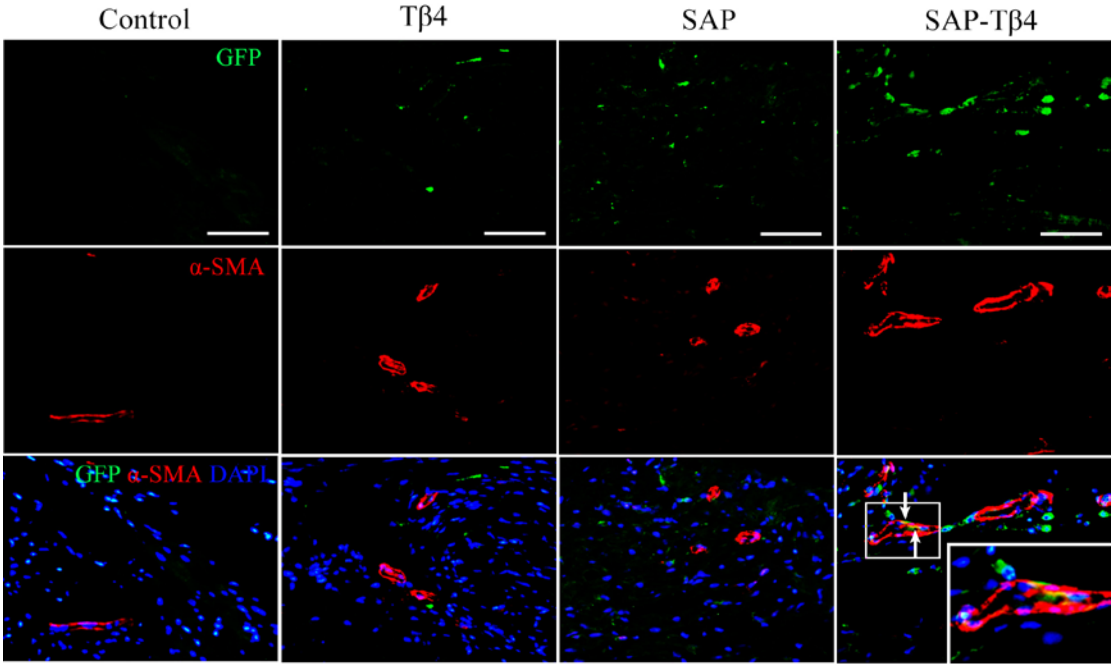

Figure S5. EPDCs differentiate into smooth muscle cells at 4 weeks after

implantation. α-SMA+ cells differentiated from EPDCs (arrows) are located at the wall of the microvessels at the infarcted region. The large box is magnification of the small box. Immunostaining. Scale bar = 100 μm.

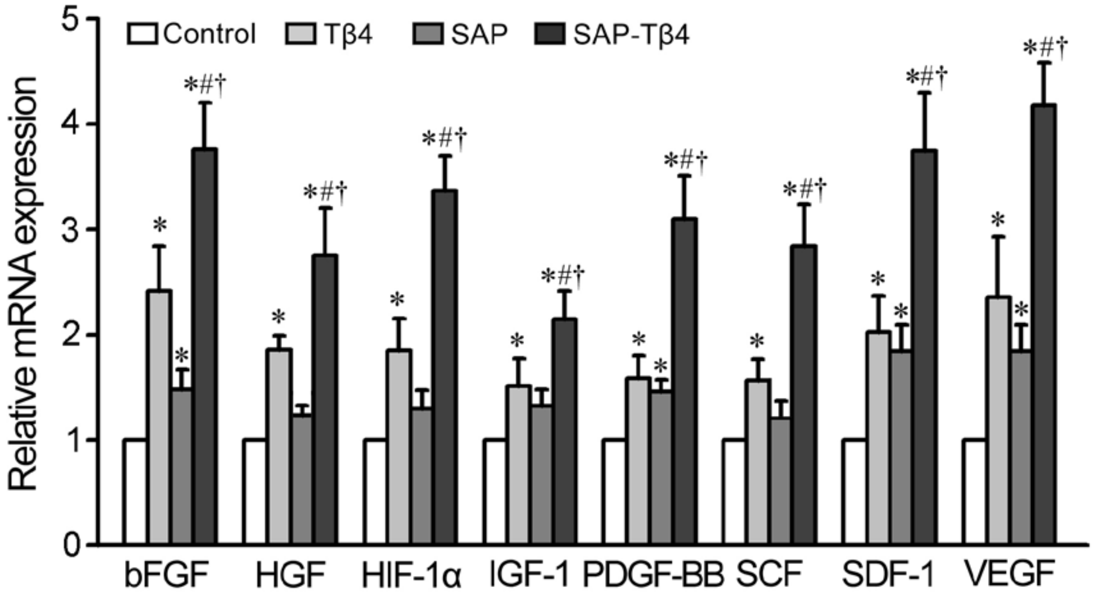

Figure S6. Expression of the genes in the infarcted myocardium at 1 week after implantation. qRT-PCR analysis. **p* < 0.05 versus control group; #*p* < 0.05 versus Tβ4 group; †*p* < 0.05 versus SAP group. n = 4.
